# Environmental Toxicant Exposure and Depressive Symptoms

**DOI:** 10.1001/jamanetworkopen.2024.20259

**Published:** 2024-07-03

**Authors:** Jianhui Guo, Eric Garshick, Feifei Si, Ziqi Tang, Xinyao Lian, Yaqi Wang, Jing Li, Petros Koutrakis

**Affiliations:** 1 Institute of Child and Adolescent Health, School of Public Health, Peking University, Beijing, China; 2 Pulmonary, Allergy, Sleep, and Critical Care Medicine Section, Medical Service, Veterans Affairs Boston Healthcare System and Harvard Medical School, Boston, Massachusetts; 3 Peking University Sixth Hospital Institute of Mental Health, NHC Key Laboratory of Mental Health (Peking University), National Clinical Research Center for Mental Disorders (Peking University Sixth Hospital), Beijing, China; 4 Department of Environmental Health, Harvard T. H. Chan School of Public Health, Boston, Massachusetts

## Abstract

**Importance:**

Recognizing associations between exposure to common environmental toxicants and mental disorders such as depression is crucial for guiding targeted mechanism research and the initiation of disease prevention efforts.

**Objectives:**

To comprehensively screen and assess the associations between potential environmental toxicants and depressive symptoms and to assess whether systemic inflammation serves as a mediator.

**Design, Setting, and Participants:**

A total of 3427 participants from the 2013-2014 and 2015-2016 waves of the National Health and Nutrition Examination and Survey who had information on blood or urine concentrations of environmental toxicants and depression scores assessed by the 9-item Patient Health Questionnaire (PHQ-9) were included. Statistical analysis was performed from July 1, 2023, to January 31, 2024.

**Exposures:**

Sixty-two toxicants in 10 categories included acrylamide, arsenic, ethylene oxide, formaldehyde, iodine, metals, nicotine metabolites, polycyclic aromatic hydrocarbons, volatile organic compound (VOC) metabolites; and perchlorate, nitrate, and thiocyanate.

**Main Outcomes and Measures:**

An exposome-wide association study and the deletion-substitution-addition algorithm were used to assess associations with depression scores (PHQ-9 ≥5) adjusted for other important covariates. A mediation analysis framework was used to evaluate the mediating role of systemic inflammation assessed by the peripheral white blood cell count.

**Results:**

Among the 3427 adults included, 1735 (50.6%) were women, 2683 (78.3%) were younger than 65 years, and 744 (21.7%) were 65 years or older, with 839 (24.5%) having depressive symptoms. In terms of race and ethnicity, 570 participants (16.6%) were Mexican American, 679 (19.8%) were non-Hispanic Black, and 1314 (38.3%) were non-Hispanic White. We identified associations between 27 chemical compounds or metals in 6 of 10 categories of environmental toxicants and the prevalence of depressive symptoms, including the VOC metabolites *N*-acetyl-*S*-(2-hydroxy-3-butenyl)-l-cysteine (odds ratio [OR], 1.74 [95% CI, 1.38, 2.18]) and total nicotine equivalent-2 (OR, 1.42 [95% CI, 1.26-1.59]). Men and younger individuals appear more vulnerable to environmental toxicants than women and older individuals. Peripheral white blood cell count mediated 5% to 19% of the associations.

**Conclusions and Relevance:**

In this representative cross-sectional study of adults with environmental toxicant exposures, 6 categories of environmental toxicants were associated with depressive symptoms with mediation by systemic inflammation. This research provides insight into selecting environmental targets for mechanistic research into the causes of depression and facilitating efforts to reduce environmental exposures.

## Key Points

**Question:**

Is there an association between common environmental toxicants and depressive symptoms among US adults?

**Findings:**

This cross-sectional study of 3427 adults from the National Health and Nutrition Examination and Survey found that 27 environmental toxicant levels measured in blood or urine samples were associated with depressive symptoms, as assessed by the 9-item Patient Health Questionnaire. Systemic inflammation assessed by peripheral white blood cell count mediated these associations.

**Meaning:**

These findings suggest that many common environmental toxicants are associated with depressive symptoms, providing potential targets for intervention measures and mechanistic research.

## Teaser

This cross-sectional study examines the association between environmental toxicant exposure and depressive symptoms in a population of US adults using an exposome approach based on data from the National Health and Nutrition Examination Survey.

## Introduction

Depression is one of the key diseases covered by the World Health Organization’s Mental Health Gap Action Programme. An estimated 3.8% of the population is affected by depression globally.^1,2^ Mental illnesses have led to the loss of several billion dollars annually in the US. The international community has invested in a concerted effort to improve mental health, but satisfactory outcomes have yet to be achieved as of 2020.^3^ Therefore, the identification of modifiable risk factors for depression is critical.^4^

Psychiatric disorders have complex, multifaceted, and interrelated environmental origins.^5^ With increasing environmental pollution, many pollutants may impact human disease.^6^ Previous studies focused on the use of a priori knowledge to assess associations between individual or single categories of candidate exposures and depression^7^ and have obtained sufficient evidence that hundreds of natural or synthetic toxicants, such as volatile organic compounds (VOCs), metals, and polycyclic aromatic hydrocarbons (PAHs), are associated with depression in humans.^8,9,10,11,12^ However, there are inherent limitations to this approach. For example, some toxicants that show an association with depression when studied in isolation may not prove robust or as clinically relevant when considered in combination with other toxicants.^13^

Exposome studies provide a scientific framework to uncover the biological consequences of exposure to a wide range of risk factors, allowing for the avoidance of problems related to selective reporting and confounding by coexposures.^14,15^ To date, exposome studies have uncovered the associations of multiple behavioral risk factors and social factors with depression,^16,17^ while exposome studies exploring the associations of environmental toxicants with depression remain limited.^8^ In addition, exploration of the associations of environmental toxicants with depressive disorders has been limited by sample size and the lack of comprehensive measurements of environmental toxicants. Therefore, a comprehensive exposome analysis of environmental toxicants is essential for investigating the association between these toxicants and depression.

Previous studies have identified the activation of inflammatory pathways as one of the key mechanisms involved in the pathogenesis of depression and have revealed several inflammatory biomarkers associated with depression, including total white blood cells (WBCs) and C-reactive protein.^18,19^ Previous studies indicate that inflammation could serve as a crucial link between environmental toxicants and depression.^20,21,22,23^ However, there are significant knowledge gaps in addressing multivariate mediation involving not only high-dimensional mediators but also multiple toxicants with inherent covariance and grouping structures. Therefore, a framework for the analysis of high-dimensional mediators is required to explore a mixed mediation setting involving multiple exposure categories and groups of endogenous biomarker mediators.^24,25^

Based on a no-hypothesis approach, the purpose of this study was to comprehensively examine the association between environmental toxicant exposure and depressive symptoms in a population of US adults using an exposome approach based on data from the National Health and Nutrition Examination Survey (NHANES) to provide a theoretical basis for policy-driven exposure reduction and depression prevention interventions. In addition, a mediation analysis framework was implemented to examine the mediating function of systemic inflammation.

## Methods

### Study Design and Dataset Generation

NHANES is a nationally representative, cross-sectional study that collects health survey data from the US resident and ambulatory civilian population, with a questionnaire administered in the home followed immediately by a standardized health survey administered in a specially equipped mobile examination center.^26^ In this study, we included survey results from 3427 participants who had environmental toxicants characterized and who completed the 9-item Patient Health Questionnaire (PHQ-9) in the 2013-2014 and 2015-2016 NHANES waves, representing approximately 240 million US adults 18 years and older (eMethods and eFigure 1 in Supplement 1).

Race and ethnicity were categorized as Mexican American, non-Hispanic Black, non-Hispanic White, other Hispanic, and other race (which comprised participants who identified as non-Hispanic multiracial). These data were collected as a confounding factor owing to the differences among individuals of different races and ethnicities regarding susceptibility to depression and ability to metabolize environmental toxins.

NHANES was approved by the Research Ethics Review Board of the US Centers for Disease Control and Prevention National Center for Health Statistics, and written informed consent was obtained from all adult participants. We followed the Strengthening the Reporting of Observational Studies in Epidemiology (STROBE) reporting guideline for cross-sectional studies.

### Outcome Definition

The PHQ-9, a depression screening instrument, was administered to determine the frequency of depressive symptoms over the past 2 weeks. The instrument incorporates *Diagnostic and Statistical Manual of Mental Disorders* (Fourth Edition) depression diagnostic criteria.^27^ Responses were categorized as “not at all,” “several days,” “more than half the days,” or “nearly every day,” with scores ranging from 0 to 3. The total score is based on the sum of the points for each item and ranges from 0 to 27. Participants can be classified into 4 categories of depression based on the questionnaire scores: asymptomatic (0-4), mild (5-9), moderate (10-14), moderately severe (15-19), and severe (20-27). We considered a score of 5 or greater to be positive for depression, but additionally grouped the participants based on PHQ-9 scores of at least 10 and at least 15 for sensitivity analyses.

### Exposure Definition

In this study, a total of 89 environmental toxicants in 13 categories were measured in the participants who were selected by NHANES investigators based on the subsample A weights. After the questionnaire survey, blood and urine samples were collected from the participants for the evaluation of the exposure level of environmental toxicants. Toxicants were assessed by either the measurement of hemoglobin adducts for reactive organic compounds such as acrylamide, glycidamide, ethylene oxide, and formaldehyde or by assaying inorganic elemental toxicants and metals in the urine. Detailed information and measurement methods for the 89 environmental toxicants and the contaminants included in each toxicant category are available in the eMethods and eTable 1 in Supplement 1. Following consideration of missing data and values below the lower limit of detection (eMethods in Supplement 1), a total of 62 types in 10 categories of environmental toxicants were included in the primary analysis. The concentrations of hemoglobin adduct were expressed as the number per milligram of hemoglobin, with analyses adjusted for hemoglobin concentration. The urine toxicants were adjusted per milligram of urine creatine to adjust for urinary dilution.

### Statistical Analysis

Data were analyzed from July 1, 2023, to January 31, 2024. We performed statistical analyses of the exposome using 2 methods, including the exposome-wide association study (ExWAS), which considers the effects of each environmental toxicant independently, and the deletion-substitution-addition (DSA) algorithm, which considers all environmental toxicants simultaneously.^28^ More details are described in the eMethods in Supplement 1. We conducted sensitivity ExWAS analyses to enhance the robustness of our findings. First, we performed sensitivity analyses based on different cutoff points (≥10 and ≥15) of the PHQ-9. We also conducted ExWAS analysis based on the data that did not impute missing values and did not exclude participants with missing values for more than one-third of all environmental toxicants.

We developed a mediation analysis framework consisting of 2 analyses, with the objective of examining whether the associations between environmental toxicants and depressive symptoms were mediated by natural log-transformed total WBC count as a surrogate for systemic inflammation. The initial analysis performed was a 1-way pairwise mediation, while the second method involved exposure dimension reduction followed by pairwise mediation analysis. Additionally, a reverse mediation analysis was conducted to confirm the absence of reverse mediation. Exposure dimension reduction followed by pairwise mediation analysis is another approach to mediation analysis that aims to reduce the dimensionality of exposures to reduce the number of mediating role models. The approach of the second option is to use exposure-class risk scores (ERS) for mediation analysis. More details about the mediation analysis framework are available in the eMethods in Supplement 1.

We included covariates in the ExWAS, DSA algorithm, and mediation analysis framework based on previous research, consisting of sex, age, race and Hispanic ethnicity, educational level, the ratio of family income to poverty, body mass index, sleep duration, alcohol consumption, estimated glomerular filtration rate, sample collection time, 6-month survey period, and survey cycles. We used the rexposome package to conduct ExWAS analyses, the dsa package for the DSA algorithm, the mediation package to analyze the mediation effects, and the *gcdnet* package for adaptive elastic net regularization. The DSA package was executed in R, version 2.15.3 (R Project for Statistical Computing), while the remaining analyses were performed using R, version 4.3.1. Two-sided *P* < .05 indicated statistical significance.

## Results

Among the 3427 participants, 1692 (49.4%) were men, 1735 (50.6%) were women, 2683 (78.3%) were younger than 65 years, and 744 (21.7%) were 65 years or older. In terms of race and ethnicity, 570 participants (16.6%) were Mexican American, 679 (19.8%) were non-Hispanic Black, 1314 (38.3%) were non-Hispanic White, 382 (11.1%) were other Hispanic, and 482 (14.1%) were other race. During the 2013-2014 survey period, the crude prevalence of depression was 427 of 1754 participants (24.3%), and during the 2015-2016 survey period, 412 of 1673 (24.6%), for an overall prevalence of 839 of 3427 (24.5%) (Table 1). The baseline data before the imputation of missing values are shown in eTable 2 in Supplement 1 and have a distribution similar to the results before the interpolation process.

**Table 1.** Participant Characteristics

Characteristic	Participant group, No. (%)		
	All (N = 3427)	No depression (n = 2588)	Depression (n = 839)
Sex			
Men	1692 (49.4)	1343 (51.9)	349 (41.6)
Women	1735 (50.6)	1245 (48.1)	490 (58.4)
Age, y			
<65	2683 (78.3)	2037 (78.7)	646 (77.0)
≥65	744 (21.7)	551 (21.3)	193 (23.0)
Race and ethnic origin			
Mexican American	570 (16.6)	440 (17.0)	130 (15.5)
Non-Hispanic Black	679 (19.8)	493 (19.0)	186 (22.2)
Non-Hispanic White	1314 (38.3)	988 (38.2)	326 (38.9)
Other Hispanic	382 (11.1)	284 (11.0)	98 (11.7)
Other race^a^	482 (14.1)	383 (14.8)	99 (11.8)
Educational level			
Less than 9th grade	371 (10.8)	263 (10.2)	108 (12.9)
9th-11th grade	405 (11.8)	266 (10.3)	139 (16.6)
High school graduate, GED, or equivalent	799 (23.3)	592 (22.9)	207 (24.7)
Some college or AA degree	1852 (54.0)	1467 (56.7)	385 (45.9)
Ratio of family income to poverty			
<1.07	899 (26.2)	600 (23.2)	299 (35.6)
1.07 to <2.06	834 (24.3)	622 (24.0)	212 (25.3)
2.06 to <3.93	845 (24.7)	651 (25.2)	194 (23.1)
≥3.93	849 (24.8)	715 (27.6)	134 (16.0)
BMI			
<25	1020 (29.8)	783 (30.3)	237 (28.2)
25 to <30	1094 (31.9)	857 (33.1)	237 (28.2)
≥30	1313 (38.3)	948 (36.6)	365 (43.5)
Sleep duration, h			
6-8	2372 (69.2)	1871 (72.3)	501 (59.7)
<6	381 (11.1)	230 (8.9)	151 (18.0)
>8	674 (19.7)	487 (18.8)	187 (22.3)
Alcohol consumption			
Yes	2402 (70.1)	1802 (69.6)	600 (71.5)
No	1025 (29.9)	786 (30.4)	239 (28.5)
eGFR, mL/min/1.73 m^2^			
<66.56	854 (24.9)	643 (24.8)	211 (25.1)
66.56 to <84.02	855 (24.9)	666 (25.7)	189 (22.5)
84.02 to <100.18	850 (24.8)	631 (24.4)	219 (26.1)
≥100.18	868 (25.3)	648 (25.0)	220 (26.2)
Sample collection time			
Morning	1612 (47.0)	1243 (48.0)	369 (44.0)
Afternoon	1254 (36.6)	928 (35.9)	326 (38.9)
Evening	561 (16.4)	417 (16.1)	144 (17.2)
6-mo Survey period			
November 1 through April 30	1716 (50.1)	1288 (49.8)	428 (51.0)
May 1 through October 31	1711 (49.9)	1300 (50.2)	411 (49.0)
Survey cycles			
2013-2014	1754 (51.2)	1327 (51.3)	427 (50.9)
2015-2016	1673 (48.8)	1261 (48.7)	412 (49.1)

Abbreviations: AA, Associate in Arts; BMI, body mass index (calculated as weight in kilograms divided by height in meters squared); eGFR, estimated glomerular filtration rate; GED, General Educational Development.

^a^ Includes participants who identified as non-Hispanic multiracial.

Among 89 toxicants, a total of 62 with sufficient data were included. Of these, 10 were analyzed dichotomously based on detection limits, and the remaining 50 were log(ln) transformed to approximate a normal distribution (eTable 1 in Supplement 1). As shown in eFigure 2 in Supplement 1, there were different degrees of correlation among the 62 environmental toxicants investigated in this study. The correlation coefficients ranged from −0.227 to 0.988, with most toxicants having a correlation coefficient of less than 0.800.

**Figure 1.**
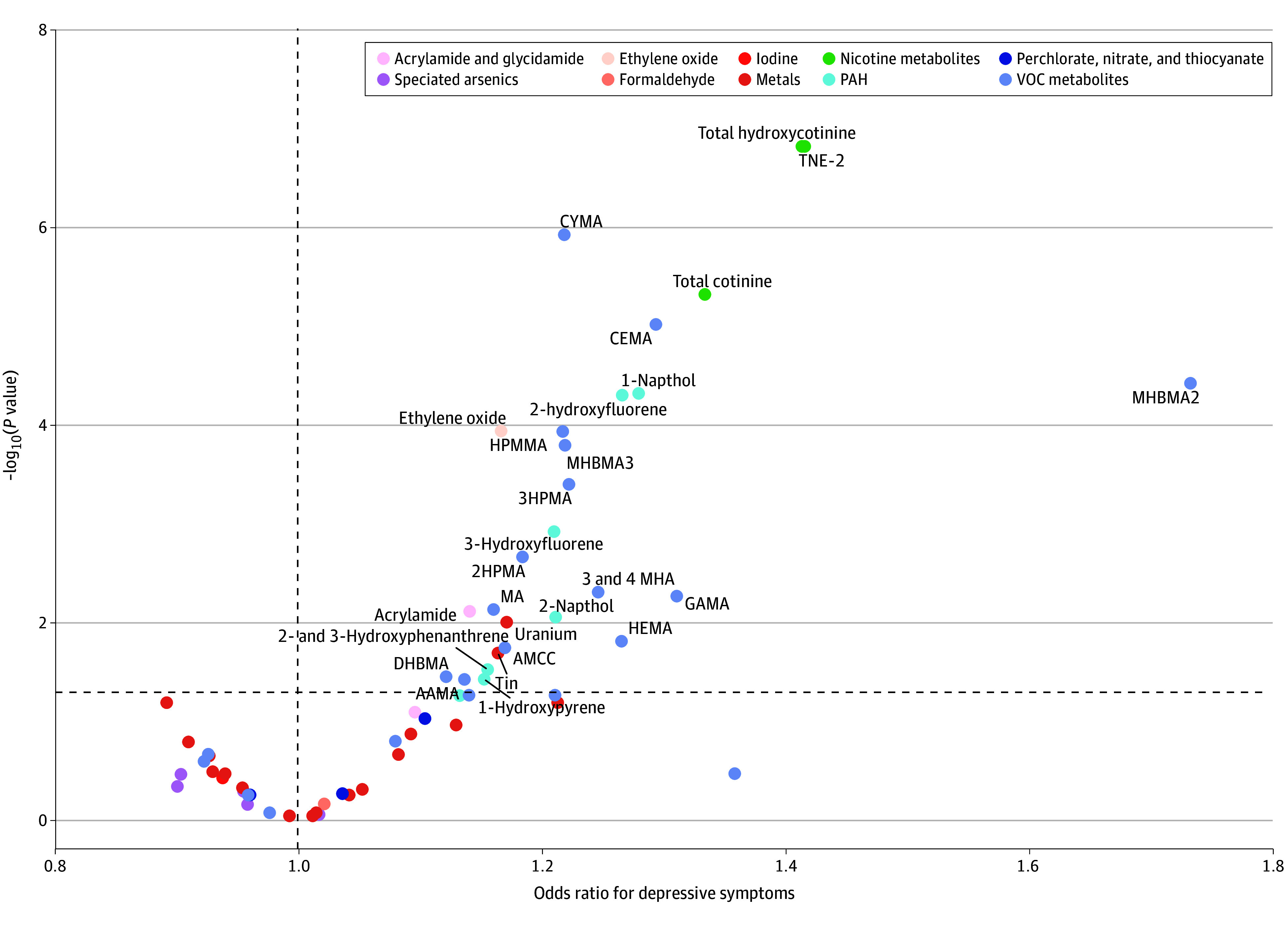
Adjusted Association Between Environmental Toxicants (62 Exposures) and Depressive Symptoms in 3427 Adults (Exposome-Wide Association Study Analysis) AAMA indicates 2-carbamoylethylmercapturic acid; AMCC, methylcarbamoylmercapturate; CEMA, cyanoethyl mercapturic acid; CYMA, *N*-acetyl-*S*-(2-cyanoethyl)-l-cysteine; DHBMA, dihydroxy-butyl-mercapturic acid; GAMA, carbamoyl-2-hydroxyethylmercapturate; HEMA, 2-hydroxyethylmercapturic acid; HPMA, hydroxypropylmercapturic acid; HPMMA, 3-hydroxy-1-methyl-propylmercapturic acid; MA, mandelic acid; MHA, methylhippuric acid; MHBMA, monohydroxybutenyl-mercapturic acid; PAH, polycyclic aromatic hydrocarbon; TNE-2, total nicotine equivalent-2; and VOC, volatile organic compound.

The ExWAS analysis accounted for potential confounding factors and revealed 27 environmental toxicants in 6 categories with a positive association with depressive symptoms. These categories include acrylamide and glycidamide, ethylene oxide, metals (2 types), nicotine metabolites (3 types), PAH (6 types), and VOC metabolites (14 types). In particular, individuals with detectable levels of MHBMA2 had a risk of depressive symptoms 1.74 (95% CI, 1.38-2.18) times higher than those with undetectable levels. Furthermore, total nicotine equivalent-2 (TNE-2) and total hydroxycotinine, which are nicotine metabolites, were also associated with depressive symptoms. For each 1-U increase in IQR, the likelihood of depressive symptoms increased by 42% (95% CI, 26%-59%) for TNE-2 and 41% (95% CI, 26%-59%) for hydroxycotinine (Table 2 and Figure 1). According to the DSA algorithm, TNE-2 and *N*-acetyl-*S*-(2-carboxyethyl)-l-cysteine (CEMA; a VOC metabolite) were identified as key factors associated with the prevalence of depressive symptoms.

**Table 2.** Associations Between Environmental Toxicants (62 Exposures) and Depressive Symptoms in 3427 Adults (ExWAS Analysis)

Exposure family by toxicant exposure	Processing	Median (IQR)	Model 1^a^		Model 2^b^	
			OR (95% CI)	*P* value^c^	OR (95% CI)	*P* value^c^
Acrylamide and glycidamide						
Acrylamide	log(ln)	42.9 (32.8-64.2)	1.18 (1.09-1.28)	<.001	1.14 (1.05-1.24)	.007
Glycidamide	log(ln)	38.4 (28.1-54.5)	1.15 (1.06-1.25)	.002	1.10 (1.01-1.20)	.07
Speciated arsenics						
Arsenobetaine	2 Categories	NA	0.97 (0.83-1.14)	.79	1.02 (0.86-1.20)	.87
Arsenocholine	2 Categories	NA	0.87 (0.70-1.07)	.22	0.90 (0.72-1.12)	.43
Arsenous acid	2 Categories	NA	0.83 (0.71-0.98)	.04	0.90 (0.76-1.07)	.34
Dimethylarsinic acid	log(ln)	3.5 (2.2-5.7)	0.95 (0.86-1.05)	.38	0.95 (0.86-1.06)	.48
Monomethylarsonic acid	2 Categories	NA	0.88 (0.75-1.04)	.17	0.95 (0.81-1.13)	.65
Ethylene oxide						
Ethylene oxide	log(ln)	21.1 (15.1-42.2)	1.23 (1.15-1.31)	<.001	1.17 (1.09-1.25)	<.001
Formaldehyde						
Formaldehyde	log(ln)	132.0 (121.0-144.0)	1.01 (0.94-1.09)	.79	1.02 (0.94-1.11)	.65
Iodine						
Iodine	log(ln)	124.3 (76.9-222.9)	0.98 (0.89-1.09)	.79	0.94 (0.84-1.05)	.37
Metals						
Copper	log(ln)	115.1 (99.0-133.6)	1.24 (1.12-1.37)	<.001	1.04 (0.93-1.17)	.55
Selenium	log(ln)	128.8 (118.9-139.8)	0.90 (0.82-0.99)	.05	0.95 (0.86-1.05)	.45
Zinc	log(ln)	80.4 (70.9-90.4)	0.88 (0.79-0.97)	.02	0.93 (0.83-1.04)	.3
Barium	log(ln)	1.1 (0.6-2.0)	0.93 (0.84-1.02)	.16	0.94 (0.85-1.04)	.34
Cadmium	log(ln)	0.2 (0.1-0.4)	1.23 (1.10-1.37)	<.001	1.13 (0.99-1.28)	.12
Cobalt	log(ln)	0.4 (0.3-0.6)	1.10 (1.00-1.21)	.08	1.02 (0.91-1.13)	.83
Cesium	log(ln)	4.2 (3.1-5.9)	0.91 (0.82-1.01)	.09	0.91 (0.81-1.01)	.14
Mercury	2 categories	NA	0.93 (0.79-1.09)	.46	1.01 (0.85-1.20)	.92
Manganese	2 categories	NA	1.32 (1.11-1.56)	.002	1.21 (1.02-1.45)	.07
Molybdenum	log(ln)	37.2 (24.8-54.4)	0.94 (0.85-1.03)	.23	0.93 (0.84-1.03)	.22
Lead	log(ln)	0.3 (0.2-0.5)	1.12 (1.01-1.24)	.06	1.05 (0.94-1.18)	.48
Antimony	log(ln)	0 (0-0.1)	1.17 (1.07-1.28)	.002	1.09 (0.99-1.21)	.13
Tin	log(ln)	0.4 (0.2-0.9)	1.29 (1.17-1.42)	<.001	1.16 (1.04-1.30)	.02
Strontium	log(ln)	96.2 (59.4-148.8)	0.99 (0.90-1.08)	.79	0.99 (0.90-1.09)	.92
Thallium	log(ln)	0.2 (0.1-0.2)	0.87 (0.79-0.96)	.008	0.89 (0.80-0.99)	.07
Tungsten	log(ln)	0.1 (0-0.1)	1.11 (1.00-1.22)	.06	1.08 (0.97-1.20)	.22
Uranium	log(ln)	0 (0-0)	1.22 (1.11-1.34)	<.001	1.17 (1.06-1.30)	.01
Nicotine metabolites						
TNE-2	log(ln)	0 (0-1.3)	1.51 (1.37-1.67)	<.001	1.42 (1.26-1.59)	<.001
Total cotinine	log(ln)	0.5 (0.2-86.1)	1.44 (1.31-1.58)	<.001	1.33 (1.20-1.49)	<.001
Total hydroxycotinine	log(ln)	0.9 (0.3-157.2)	1.51 (1.37-1.67)	<.001	1.41 (1.26-1.59)	<.001
PAH						
1-Napthol	log(ln)	1392.5 (708.1-4718.5)	1.39 (1.26-1.54)	<.001	1.28 (1.15-1.42)	<.001
2-Napthol	log(ln)	5664.4 (2898.7-10 933.4)	1.42 (1.26-1.59)	<.001	1.21 (1.07-1.37)	.008
3-Hydroxyfluorene	log(ln)	71.7 (42.5-189.7)	1.30 (1.19-1.43)	<.001	1.21 (1.09-1.34)	.001
2-Hydroxyfluorene	log(ln)	174.8 (109.8-382.4)	1.39 (1.27-1.53)	<.001	1.27 (1.14-1.40)	<.001
1-Hydroxyphenanthrene	log(ln)	104.6 (69.1-169.7)	1.22 (1.10-1.35)	<.001	1.13 (1.01-1.26)	.06
1-Hydroxypyrene	log(ln)	131.6 (80.5-225.0)	1.27 (1.14-1.41)	<.001	1.15 (1.03-1.29)	.04
2- and 3-Hydroxyphenanthrene	log(ln)	127.5 (82.8-209.0)	1.26 (1.14-1.40)	<.001	1.16 (1.03-1.29)	.03
Perchlorate, nitrate, and thiocyanate						
Nitrate	log(ln)	42 911.3 (30 973.3-63 739.6)	1.01 (0.92-1.10)	.92	1.04 (0.95-1.14)	.52
Thiocyanate	log(ln)	1049.0 (532.2-2212.7)	1.18 (1.07-1.29)	.002	1.10 (1.00-1.22)	.10
Perchlorate	log(ln)	2.6 (1.6-4.3)	0.96 (0.87-1.07)	.54	0.96 (0.86-1.07)	.54
VOC metabolites						
2,2DCVMA	2 categories	NA	1.04 (0.89-1.22)	.66	1.35 (0.80-2.27)	.34
2MHA	log(ln)	31.8 (14.8-73.1)	1.16 (1.04-1.29)	.01	1.14 (1.02-1.28)	.05
3 and 4 MHA	log(ln)	189.7 (94.1-508.0)	1.29 (1.14-1.45)	<.001	1.25 (1.09-1.42)	.005
AAMA	log(ln)	50.9 (31.8-90.0)	1.20 (1.09-1.32)	<.001	1.14 (1.02-1.26)	.04
AMCC	log(ln)	145.1 (78.3-262.9)	1.29 (1.16-1.43)	<.001	1.17 (1.04-1.31)	.02
ATCA	log(ln)	121.9 (60.8-227.2)	1.09 (0.98-1.21)	.13	0.96 (0.85-1.08)	.55
BMA	log(ln)	6.5 (4.0-11.4)	0.98 (0.89-1.07)	.67	0.92 (0.84-1.02)	.19
BPMA	log(ln)	3.9 (1.7-9.8)	0.89 (0.79-0.99)	.05	0.92 (0.82-1.03)	.24
CEMA	log(ln)	97.2 (61.9-161.1)	1.40 (1.27-1.54)	<.001	1.29 (1.17-1.43)	<.001
CYMA	log(ln)	1.7 (1.0-8.7)	1.28 (1.20-1.37)	<.001	1.22 (1.14-1.31)	<.001
DHBMA	log(ln)	303.3 (232.3-400.9)	1.20 (1.10-1.30)	<.001	1.12 (1.02-1.23)	.04
GAMA	2 Categories	NA	1.37 (1.17-1.60)	<.001	1.31 (1.11-1.55)	.005
HEMA	2 Categories	NA	1.35 (1.16-1.58)	<.001	1.27 (1.08-1.50)	.01
2HPMA	log(ln)	27.8 (17.7-51.3)	1.21 (1.10-1.32)	<.001	1.19 (1.08-1.30)	.002
3HPMA	log(ln)	238.0 (146.2-460.5)	1.28 (1.17-1.40)	<.001	1.22 (1.11-1.35)	<.001
MA	log(ln)	133.0 (93.0-196.2)	1.25 (1.14-1.36)	<.001	1.16 (1.06-1.28)	.007
MHBMA2	2 Categories	NA	2.02 (1.63-2.50)	<.001	1.74 (1.38-2.18)	<.001
MHBMA3	log(ln)	4.7 (2.9-9.6)	1.31 (1.20-1.43)	<.001	1.22 (1.11-1.34)	<.001
PHEMA	2 Categories	NA	1.35 (1.16-1.58)	<.001	1.21 (1.03-1.43)	.05
PGA	log(ln)	201.7 (144.6-289.9)	1.15 (1.06-1.26)	.002	1.08 (0.99-1.18)	.16
PMA	2 Categories	NA	1.00 (0.85-1.17)	.97	0.98 (0.83-1.16)	.83
HPMMA	log(ln)	214.3 (148.7-427.0)	1.31 (1.21-1.43)	<.001	1.22 (1.11-1.33)	<.001

Abbreviations: 2,2DCVMA, *N*-acetyl-*S*-(2,2-dichlorovinyl)-l-cysteine; AAMA, 2-carbamoylethylmercapturic acid; AMCC, methylcarbamoylmercapturate; ATCA, 2-aminothiazoline-4-carboxylic acid; BMA, benzylmercapturic acid; BPMA, *N*-acetyl-*S*-(n-propyl)-l-cysteine; CEMA, *N*-acetyl-*S*-(2-carboxyethyl)-l-cysteine; CYMA, *N*-acetyl-*S*-(2-cyanoethyl)-l-cysteine; DHBMA, dihydroxy-butyl-mercapturic acid; ExWAS, exposome-wide association study; GAMA, carbamoyl-2-hydroxyethylmercapturate; HEMA, 2-hydroxyethylmercapturic acid; HPMA, hydroxypropylmercapturic acid; HPMMA, 3-hydroxy-1-methyl-propylmercapturic acid; MA, mandelic acid; MHA, methylhippuric acid; MHBMA, *N*-acetyl-*S*-(2-hydroxy-3-butenyl)-l-cysteine; NA, not applicable; OR, odds ratio; PAH, polycyclic aromatic hydrocarbon; PGA, polyglutamic acid; PHEMA, poly(2-hydroxyethyl methacrylate); PMA, phenylmercapturic acid; TNE-2, total nicotine equivalent-2; VOC, volatile organic compound.

^a^ Unadjusted model.

^b^ Constructed by adjusting for sex, age, race and Hispanic ethnicity, educational level, the ratio of family income to poverty, body mass index, sleep duration, alcohol consumption, estimated glomerular filtration rate, sample collection time, 6-month survey period, and survey cycles.

^c^ Adjusted to control the false discovery rate at 5%.

**Figure 2.**
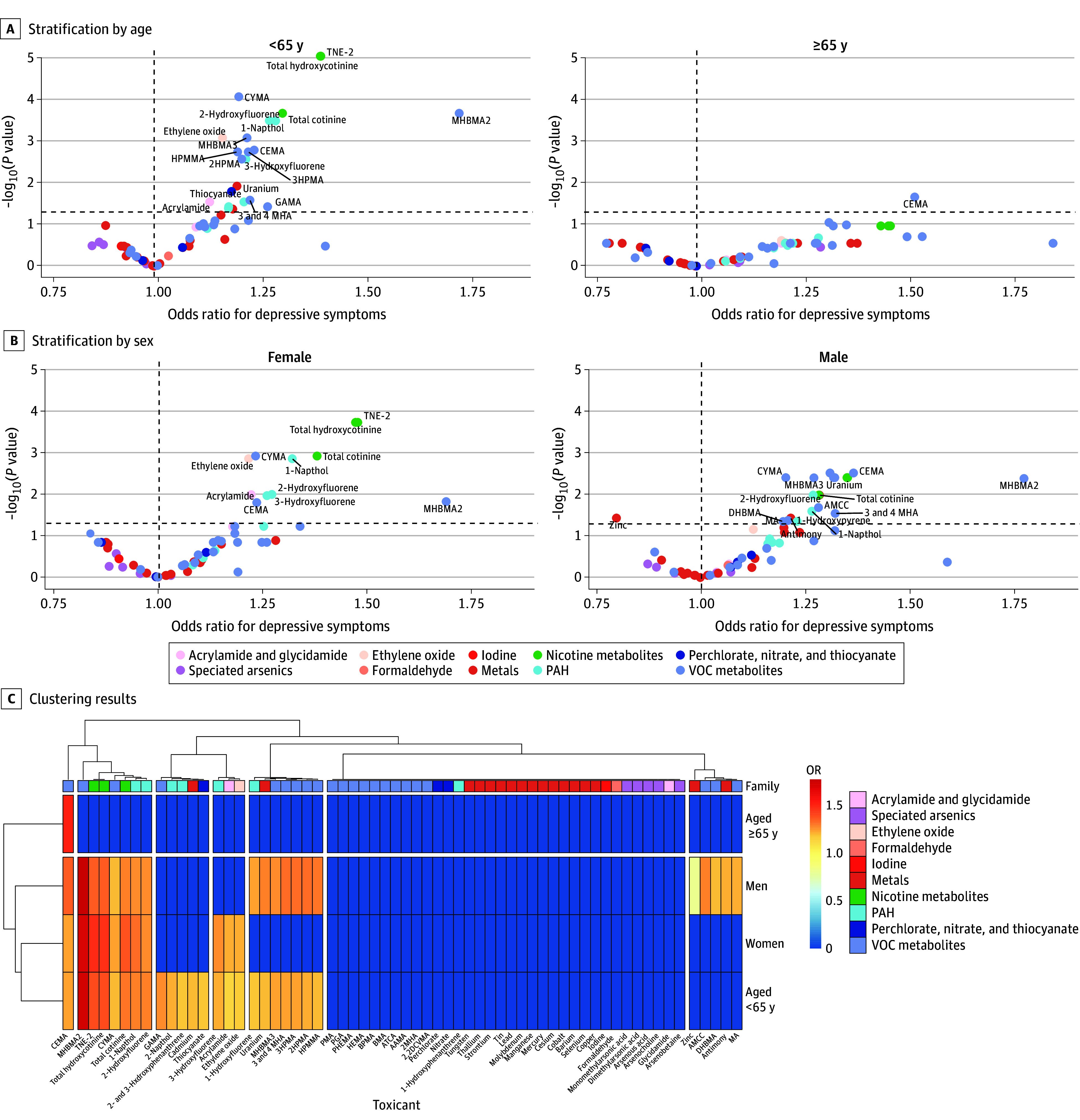
Adjusted Association Between Environmental Toxicants (62 Exposures) and Depressive Symptoms in 3427 Adults (Exposome-Wide Association Study Analysis) Stratified by Age and Sex 2,2DCVMA indicates *N*-acetyl-*S*-(2,2-dichlorovinyl)-l-cysteine; AAMA, 2-carbamoylethylmercapturic acid; AMCC, methylcarbamoylmercapturate; ATCA, 2-aminothiazoline-4-carboxylic acid; BMA, benzylmercapturic acid; BPMA, *N*-acetyl-*S*-(n-propyl)-l-cysteine; CEMA, cyanoethyl mercapturic acid; CYMA, *N*-acetyl-*S*-(2-cyanoethyl)-l-cysteine; DHBMA, dihydroxy-butyl-mercapturic acid; GAMA, carbamoyl-2-hydroxyethylmercapturate; HEMA, 2-hydroxyethylmercapturic acid; HPMA, hydroxypropylmercapturic acid; HPMMA, 3-hydroxy-1-methyl-propylmercapturic acid; MA, mandelic acid; MHA, methylhippuric acid; MHBMA, monohydroxybutenyl-mercapturic acid; PAH, polycyclic aromatic hydrocarbon; PGA, polyglutamic acid; PHEMA, poly(2-hydroxyethyl methacrylate); PMA, phenylmercapturic acid, TNE-2, total nicotine equivalent-2; and VOC, volatile organic compound.

The analysis was stratified by age and sex. The findings suggest that more types of environmental toxicants were associated with depressive symptoms among men (20 toxicants) and individuals younger than 65 years (23 toxicants) compared with women and individuals 65 years or older, particularly for nicotine and VOC metabolites (Figure 2 and eTable 3 in Supplement 1).

Sensitivity analyses were conducted to confirm the robustness of our observed connections. Using PHQ-9 scores of 10 or more and 15 or more as cutoff values for grouping, our findings revealed that when using a PHQ-9 score of 10 or more as the cutoff, a total of 23 environmental toxicants across 7 categories were associated with depressive symptoms. When the cutoff was increased to a PHQ-9 score of 15 or more, 8 environmental toxicants across 3 categories remained associated with depressive symptoms. Notably, 4 environmental toxicants across 2 categories were consistently associated with depressive symptoms, regardless of the cutoff value, including nicotine metabolites and thallium. It is noteworthy that some environmental toxicants, such as thallium and arsenocholine, were negatively correlated with depressive symptoms at increasing PHQ-9 cutoff values. Furthermore, MHBMA2 continued to be associated with depressive symptoms (eFigure 3 and eTable 4 in Supplement 1). In addition, we also conducted an ExWAS analysis based on the data that did not impute missing values and did not exclude participants with missing values for more than one-third of all environmental toxicants. The results suggest that a total of 29 environmental toxicants across 6 categories were associated with depressive symptoms based on the data that did not impute missing values, and a total of 28 environmental toxicants across 6 categories were associated with depressive symptoms based on the data that did not exclude participants with missing values for more than one-third of all environmental toxicants (eFigure 4 and eTable 5 in Supplement 1).

**Figure 3.**
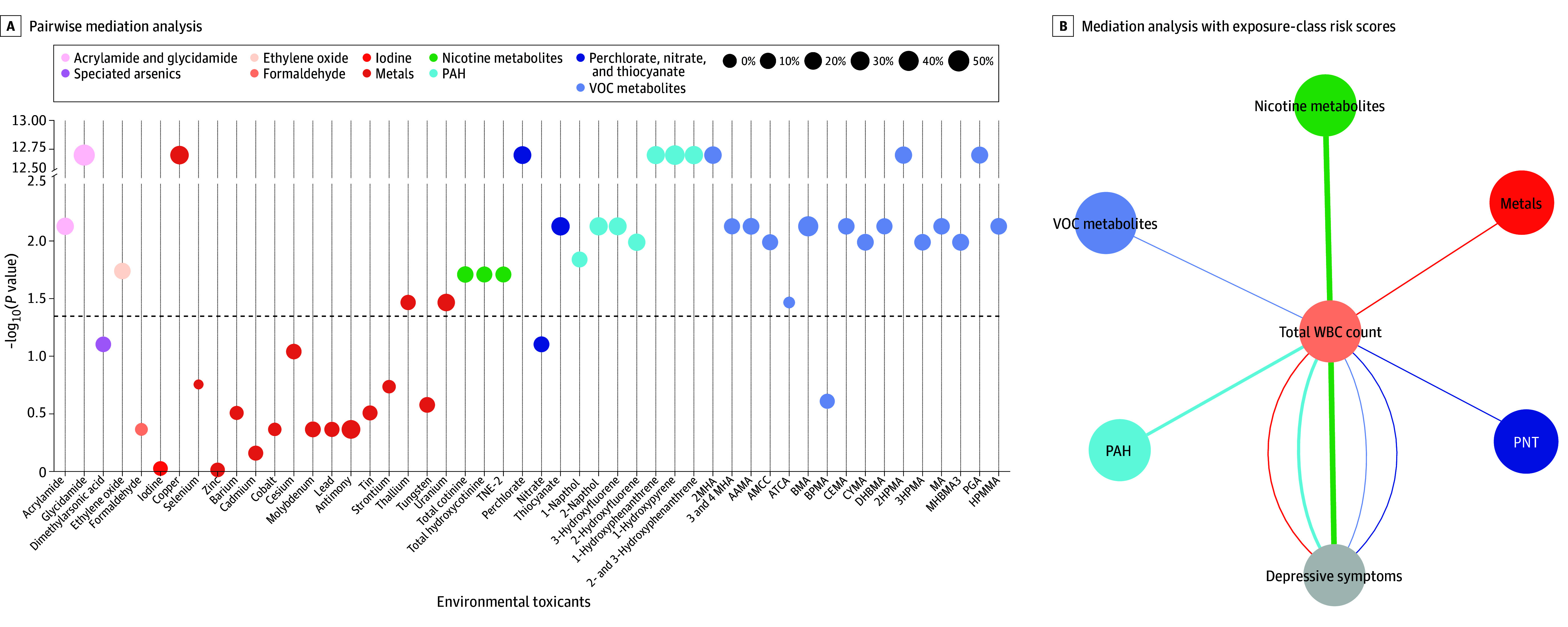
Mediation of Association Between Environmental Toxicants and Depressive Symptoms by Inflammation Biomarkers AAMA indicates 2-carbamoylethylmercapturic acid; AMCC, methylcarbamoylmercapturate; ATCA, 2-aminothiazoline-4-carboxylic acid; BMA, benzylmercapturic acid; BPMA, *N*-acetyl-*S*-(n-propyl)-l-cysteine; CEMA, cyanoethyl mercapturic acid; CYMA, *N*-acetyl-*S*-(2-cyanoethyl)-l-cysteine; DHBMA, dihydroxy-butyl-mercapturic acid; HPMA, hydroxypropylmercapturic acid; HPMMA, 3-hydroxy-1-methyl-propylmercapturic acid; MA, mandelic acid; MHBMA, monohydroxybutenyl-mercapturic acid; PAH, polycyclic aromatic hydrocarbon; PGA, polyglutamic acid; PNT, perchlorate, nitrate, and thiocyanate; TNE-2, total nicotine equivalent-2; VOC, volatile organic compound; and WBC, white blood cell.

A mediation analysis framework was used to further explore the mechanisms associated with the prevalence of depressive symptoms due to environmental toxicants. We found that total WBC count had a mediating role in associations of 33 environmental toxicants with depressive symptoms, as shown in Figure 3 and eTable 6 in Supplement 1. There were 17 direct associations between environmental toxicants and depressive symptoms in 33 mediated models, and inflammatory biomarkers mediated 5% to 19% of these associations in 17 mediated models. Reverse mediation analysis revealed that inflammatory biomarkers played a mediating role in 31 inverse associations of environmental toxicants and depressive symptoms (eFigure 5 and eTable 7 in Supplement 1) . However, for the associations of environmental toxicants and depressive symptoms, 9 intermediate models met the criterion of *P* < .05/50, while in reverse associations of environmental toxicants and depressive symptoms, none met the criteria. This suggests that the total WBC count, possibly acting as a surrogate for systemic inflammation, may mediate the association between environmental toxicants and depressive symptoms. In addition, we assessed the mediating role of total WBC count in ERS and depressive symptoms associations and found that total WBC count mediated the association of ERS (nicotine metabolites, VOC metabolites, PAHs, and metals) with depressive symptoms, as shown in Figure 3 and eTable 8 in Supplement 1.

## Discussion

In this cross-sectional profiling of environmental toxicants to uncover risk factors for depressive symptoms, we found that 6 of 10 categories of toxicants were associated with the risk of depressive symptoms. Furthermore, men and individuals younger than 65 years appear more vulnerable to environmental toxicants than women and older individuals. Systemic inflammation may mediate the association between environmental toxicants (or ERS) and depressive symptoms. This suggests that more attention should be paid to exposure to environmental toxicants in the population to reduce the risk of depression.

Our findings indicate that of the 10 categories of toxicants we surveyed, 6 were associated with the risk of depressive symptoms: nicotine metabolites, VOC metabolites, PAH, acrylamide and glycidamide, ethylene oxide, and metals. Our finding that nicotine exposure was associated with depressive symptoms is supported by previous studies.^11,29^ Although many smokers report that smoking can alleviate the negative effects of depression, a longitudinal birth cohort study found an association when it was assessed prospectively.^30^ A meta-analysis suggested that nicotine has a negative impact on childhood brain development during adolescence and may have long-term negative effects on the brain and behavior.^31,32^ In addition, our study is the first, to our knowledge, to systematically examine the association between VOC metabolites and depressive symptoms, which may reflect the metabolism of VOCs in the body and specific toxic byproducts.^12^ Our findings suggest that most of the VOC metabolites, especially MHBMA2 and CEMA, are associated with depressive symptoms, which is also consistent with previous studies.^12,33^

A previous meta-analysis^34^ revealed that PAH compounds were associated with an increase in depressive symptoms among adults, with 1-napthol showing the strongest overall association, consistent with our findings. Experimental studies have also indicated that PAH exposure can lead to brain development defects through oxidative stress and can result in specific anxiety-related behavioral disorders.^35^ In addition, previous studies have shown associations between depressive symptoms and 4 other categories of toxicants, including acrylamide,^21,36^ ethylene oxide,^37^ and heavy metals.^38,39,40,41^ Overall, ample evidence supports our exposome research on toxicants. Implementing targeted measures based on these findings could play a pivotal role in preventing and treating depression.

Sensitivity analyses showed that our results remained relatively robust. However, we found a positive association of arsenocholine and thallium with depressive symptoms, and there are several possible reasons for this. First, the use of antidepressants can lead to the development of obesity, which can cause some lipophilic environmental toxins to accumulate in adipose tissue, reducing their concentration in the blood or urine.^42^ Second, magnesium therapy can lead to rapid recovery from major depression.^43^ However, magnesium supplementation may affect the metabolism of some metals in the body, which may explain the finding in this study that exposure to thallium and cesium is protective against major depression. In addition, arsenocholine is found in seafood and is less harmful, and levels in the body can be altered by dietary changes. More evidence is needed with further studies.^44^

Furthermore, we observed differential susceptibility to toxicants across various demographic characteristics, with a greater association among men and young individuals than among women and older individuals. Previous research has shown that men may be more vulnerable to the effects of certain environmental toxicants than women due to the impact of testosterone on neurotoxicants. Women also have increased levels of glutathione and estrogen, which provide protection against environmental toxicants.^45,46,47^ In addition, young individuals may have more routes of exposure to environmental toxicants, such as occupational exposures, cosmetics, and home renovations, than older individuals.^48^ Furthermore, the brain’s development can continue until early adulthood, and during this developmental process, the brain may be more susceptible to environmental pollution.^49^ Therefore, it is crucial to pay more attention to the exposure of young people to environmental toxicants.

Environmental exposure–induced oxidative stress leading to inflammation is a critical factor contributing to a range of human diseases.^20^ Depression is associated with chronic systemic inflammation, cell-mediated immune activation, and chronic inflammation. Therefore, inflammation could act as an intermediary factor in the link between environmental chemical exposure and the onset of depression. In our study, we used a mediation analysis framework with distinct penalization and estimation algorithms for each type of analysis. However, the consistent findings throughout these methods highlight the importance of the associations, increasing our confidence in the actual mediating pathways. Using both methods, we found that inflammation plays a mediating role in the associations between depressive symptoms and VOCs, nicotine, metal, and PAH. Previous evidence also supports our findings.^22,23^ For example, imbalances in specific metal levels can weaken the structure, regulation, and catalytic functions of enzymes, proteins, receptors, and transport proteins, which, induced by oxidative stress, can lead to inflammation and decreased metal proteins, contributing to the development of various neurological disorders.^22^ Additionally, inflammation has been identified as a crucial factor linking nicotine and depression. Omega-3 fatty acids prevent nicotine withdrawal–induced exacerbation of anxiety and depression in rats by modulating oxidative stress and the inflammatory response.^23^ Further research is necessary to explore the role of inflammation in the association between other toxicants and depression.

### Strengths and Limitations

To the best of our knowledge, the present study is the largest and most recent exposome study of environmental toxicant exposure that simultaneously considers the associations between a comprehensive set (10 classes and 62 types) of toxicants and depressive symptoms. We used a no-hypothesis approach, which is characterized by the systematic testing of many variables in relation to a single outcome, to identify all toxicants associated with the development of depressive symptoms.

Our study has several limitations that warrant consideration. First, the emergence of a plethora of newly synthesized chemicals has resulted in the existence of thousands of chemical compounds.^15^ The environmental toxicants encompassed in this study represent only a fraction of this diversity, highlighting the need for further identification of environmental toxicants that may have stronger associations with depressive symptoms. Second, several environmental toxicants were excluded due to detection limits, underscoring the necessity of enhancing the sensitivity of detection instruments. Third, our cross-sectional study did not directly establish a causal relationship between environmental toxins and depressive symptoms; furthermore, assessing depressive symptoms solely through scales does not constitute a diagnosis of depression. Fourth, there may be some unadjusted confounders, such as genetic risks of depression and social factors. Fifth, despite demonstrating the unidirectionality of mediation using bidirectional mediation, which is an effective method for analyzing mediation in cross-sectional studies,^50^ prospective research is also required for additional validation. Finally, instead of using inflammatory biomarkers directly, the total WBC count was used as a surrogate for systemic inflammation in this study.

## Conclusions

In this cross-sectional study, a total of 27 environmental toxicants in 6 categories were found to be associated with the prevalence of depressive symptoms. The susceptibility to environmental toxicants varied across populations, especially among men and individuals younger than 65 years, compared with women and older individuals. Systemic inflammation, as assessed by the total WBC, may mediate multiple associations of environmental toxicants and depressive symptoms. This research highlights the significance of preventing and regulating important environmental toxicants to gain fresh insights into preventing and potentially treating depression.

## Notes

Supplement 1. **eMethods.** Data Collection, Toxicant Detection, and Analysis **eTable 1.** Characteristics of 89 Environmental Contaminants or Metabolites of Environmental Contaminants **eTable 2.** Characterization of Participants in This Study Before Data Interpolation **eTable 3.** Adjusted Association Between Environmental Toxicants (62 Exposures) and Depressive Symptoms in 3427 Adults (ExWAS Analysis) Stratified by Age and Sex **eTable 4.** Adjusted Association Between Environmental Toxicants (62 Exposures) and Depressive Symptoms in 3427 Adults (ExWAS Analysis), Compared With Threshold-Based Results **eTable 5.** Sensitivity Analyses of the Adjusted Associations Between the Environmental Toxicants (62 Exposures) and Depressive Symptoms (ExWAS Analysis) **eTable 6.** Mediation of Association Between Environmental Toxicants and Depressive Symptoms by Inflammation Biomarkers **eTable 7.** Mediation of the Inverse Association Between Environmental Toxicants and Depressive Symptoms by Inflammation Biomarkers **eTable 8.** Mediation of the Association Between Exposure-Class Risk Score (ERS) and Depressive Symptoms by Inflammation Biomarkers **eFigure 1.** Flowchart of Sample Selection **eFigure 2.** Correlation of the Environmental Toxicants (62 Exposures) **eFigure 3.** Adjusted Associations Between the Environmental Exposome (62 Exposures) and Depressive Symptoms in 3427 Adults (ExWAS Analysis), Compared With Threshold-Based Results **eFigure 4.** Sensitivity Analyses of Adjusted Associations Between the Environmental Exposome (62 Exposures) and Depressive Symptoms (ExWAS Analysis), Compared With Threshold-Based Results **eFigure 5.** Mediation of the Inverse Association Between Environmental Toxicants and Depressive Symptoms by Total WBC by Pairwise Mediation Analysis
eReferences

Supplement 2.
Data Sharing Statement
