# Supplementary material for: Environmental Toxicant Exposure and Depressive Symptoms
Source: JAMA Netw Open. 2024 Jul 3;7(7):e2420259. doi: 10.1001/jamanetworkopen.2024.20259 (PMC11222999; doi:10.1001/jamanetworkopen.2024.20259)
Supplement: Supplement 1. — eMethods. Data Collection, Toxicant Detection, and Analysis eTable 1. Characteristics of 89 Environmental Contaminants or Metabolites of Environmental Contaminants eTable 2. Characterization of Participants in This Study Before Data Interpolation eTable 3. Adjusted Association Between Environmental Toxicants (62 Exposures) and Depressive Symptoms in 3427 Adults (ExWAS Analysis) Stratified by Age and Sex eTable 4. Adjusted Association Between Environmental Toxicants (62 Exposures) and Depressive Symptoms in 3427 Adults (ExWAS Analysis), Compared With Threshold-Based Results eTable 5. Sensitivity Analyses of the Adjusted Associations Between the Environmental Toxicants (62 Exposures) and Depressive Symptoms (ExWAS Analysis) eTable 6. Mediation of Association Between Environmental Toxicants and Depressive Symptoms by Inflammation Biomarkers eTable 7. Mediation of the Inverse Association Between Environmental Toxicants and Depressive Symptoms by Inflammation Biomarkers eTable 8. Mediation of the Association Between Exposure-Class Risk Score (ERS) and Depressive Symptoms by Inflammation Biomarkers eFigure 1. Flowchart of Sample Selection eFigure 2. Correlation of the Environmental Toxicants (62 Exposures) eFigure 3. Adjusted Associations Between the Environmental Exposome (62 Exposures) and Depressive Symptoms in 3427 Adults (ExWAS Analysis), Compared With Threshold-Based Results eFigure 4. Sensitivity Analyses of Adjusted Associations Between the Environmental Exposome (62 Exposures) and Depressive Symptoms (ExWAS Analysis), Compared With Threshold-Based Results eFigure 5. Mediation of the Inverse Association Between Environmental Toxicants and Depressive Symptoms by Total WBC by Pairwise Mediation Analysis eReferences [file jamanetwopen-e2420259-s001.pdf]

Supplementary Online Content

Guo J, Garshick E, Si F, et al. Environmental toxicant exposure and depressive symptoms. *JAMA Netw Open*. 2024;7(7):e2420259.
doi:10.1001/jamanetworkopen.2024.20259

eMethods. Data Collection, Toxicant Detection, and Analysis
eTable 1. Characteristics of 89 Environmental Contaminants or Metabolites of Environmental Contaminants
eTable 2. Characterization of Participants in This Study Before Data Interpolation
eTable 3. Adjusted Association Between Environmental Toxicants (62 Exposures) and Depressive Symptoms in 3427 Adults (ExWAS Analysis) Stratified by Age and Sex
eTable 4. Adjusted Association Between Environmental Toxicants (62 Exposures) and Depressive Symptoms in 3427 Adults (ExWAS Analysis), Compared With Threshold-Based Results
eTable 5. Sensitivity Analyses of the Adjusted Associations Between the Environmental Toxicants (62 Exposures) and Depressive Symptoms (ExWAS Analysis)
eTable 6. Mediation of Association Between Environmental Toxicants and Depressive Symptoms by Inflammation Biomarkers
eTable 7. Mediation of the Inverse Association Between Environmental Toxicants and Depressive Symptoms by Inflammation Biomarkers
eTable 8. Mediation of the Association Between Exposure-Class Risk Score (ERS) and Depressive Symptoms by Inflammation Biomarkers
eFigure 1. Flowchart of Sample Selection
eFigure 2. eFigure 2. Correlation of the Environmental Toxicants (62 Exposures)
eFigure 3. Adjusted Associations Between the Environmental Exposome (62 Exposures) and Depressive Symptoms in 3427 Adults (ExWAS Analysis), Compared With Threshold-Based Results
eFigure 4. Sensitivity Analyses of Adjusted Associations Between the Environmental Exposome (62 Exposures) and Depressive Symptoms (ExWAS Analysis), Compared With Threshold-Based Results
eFigure 5. Mediation of the Inverse Association Between Environmental Toxicants and Depressive Symptoms by Total WBC by Pairwise Mediation Analysis
eReferences

This supplementary material has been provided by the authors to give readers additional information about their work.

© 2024 Guo J et al. *JAMA Network Open*.

eMethods. Data Collection, Toxicant Detection, and Analysis

1. Data Collection Procedures for the National Health and Nutrition Examination and Survey

NHANES uses a complex, multistage probability design to sample the civilian, noninstitutionalized population residing in the 50 states and D.C. Sample selection for NHANES followed these stages, in order: 1) Selection of primary sampling units (PSUs), which are Sample selection for NHANES followed these stages, in order: 1) Selection of primary sampling units (PSUs), which are counties or small groups of contiguous counties. 2) Selection of segments within PSUs that constitute a block or group of blocks containing a cluster of households. 3) Selection of segments within PSUs that constitute a block or group of blocks containing a cluster of households. 4) Selection of specific households within segments. 5) Selection of individuals within a household. In 2013-2014, 14,332 persons were selected for NHANES from 30 different survey locations. In 2015-2016, 15,327 persons were selected for NHANES from 30 different survey locations. Some NHANES components were collected or processed on a subset sample (subsample) of individuals. Subsampling was performed to reduce participant burden and facilitate the scheduling and completion of the survey. Subsampling was performed to reduce participant burden and facilitate the scheduling and completion of examinations. Each subsample was selected to constitute a nationally representative sample.

2. Detection methods for environmental toxicants

Thirteen categories of toxicants were examined in this study. Detection of the toxicants was conducted using standardized assays described in NHANES documentation and briefly described below.[1,2]

2.1. Environmental toxicants in blood

Acrylamide and glycidamide: Acrylamide (AA) has been recognized as having neurotoxic, mutagenic, and potential genotoxic effects on both animals and humans. Exposure to acrylamide can occur through various occupational processes, such as those found in wastewater treatment, ore processing, paper and textile industries, and the manufacturing of dyes and adhesives. Additionally, acrylamide exposure can result from smoking tobacco and the consumption of dry, hot foods. Glycidylamide (GA) is the primary metabolite of AA and is more likely to form adducts in vivo compared with AA due to its greater nucleophilicity. A modified Edelman reaction-based method has been developed to detect hemoglobin adducts of AA and GA in red blood cells, with results reported in pmol adduct per gram of hemoglobin. The procedure involves[3]:

1. Sample Preparation for Measuring Hemoglobin Adducts of AA and GA.

2. Determination of total haemoglobin in the sample solution used for haemoglobin adduct measurements.

3. The modified Edman reaction was implemented in the sample solution, followed by the isolation of the Edman products.

4. Analysis of Edman Products Using High-Performance Liquid Chromatography Coupled with Tandem Mass Spectrometry (HPLC-MS/MS) and Subsequent Data Analysis.

Ethylene oxide (EO) is an industrial chemical utilized for manufacturing both consumer and non-consumer products. It is also used as a gaseous sterilizing agent for medical equipment. The EO detection methods and procedures were consistent with those used for acrylamide and glycidamide. [3]

Formaldehyde is a substance found in sources such as tobacco smoke, building

© 2024 Guo J et al. *JAMA Network Open*.

materials, and furniture[4]. It is highly reactive with biomolecules and can react with proteins, resulting in the formation of "adducts", such as in hemoglobin, in red blood cells.[5] The procedure involves[6]:

1. Preparation of the specimen for measurement;

2. Total haemoglobin in the sample solution used for haemoglobin adduct measurements;

3. Enzymatic digestion of the sample solution;

4. Analysis of the digested samples by HPLC-MS/MS; and

5. HPLC–MS/MS data processing and evaluation.

2.2. Environmental toxicants in urine

Aromatic amine pollutants primarily result from tobacco combustion, as well as exposure to chemicals used in industry, such as, dyes and pigments, pharmaceuticals, pesticides, herbicides, synthetic rubbers, and plastics. Additionally, environmental pollution, including diesel exhaust, combustion of wood shavings and rubber, and substances in charcoal-grilled meat and fish, contributes to their release. Aromatic amines in urine are quantified by an isotope-dilution gas chromatographic, tandem mass spectrometric method (gas chromatography-tandem mass spectrometry).[7]

Heterocyclic aromatic amines (HCAAs) are an important class of carcinogens formed during cigarette combustion and high temperature cooking of meat. The presence of heterocyclic aromatic amines (HCAAs) in urine was determined by isotope dilution high performance liquid chromatography/electrospray ionization tandem mass spectrometry (high-performance liquid chromatography-electrospray ionization tandem mass spectrometry).

Arsenic is widespread in the Earth's crust and is typically present in groundwater rather than surface water. There are various chemical forms of arsenic that differ considerably in their toxicity. The most toxic arsenic compounds that occur naturally are inorganic forms and their methylated metabolites. Organic arsenic compounds are less toxic. The concentration of speciated arsenics in urine is determined by using high

© 2024 Guo J et al. *JAMA Network Open*.

performance liquid chromatography to separate the species coupled to an inductively coupled plasma dynamic reaction cell mass spectrometry to detect the arsenic species.[8]

Metals: Mass spectrometry can be used to directly measure multiple metals in urine samples after simple dilution. This method directly measures multiple metals in urine specimens using mass spectrometry after a simple dilution sample preparation step. Liquid samples are introduced into the mass spectrometer through the inductively coupled plasma (ICP) ionization source and reduced to small droplets in an argon aerosol via a nebulizer, after which the droplets enter the ICP. The ions first pass through a focusing region, followed by the dynamic reaction cell (DRC) and the quadrupole mass filter, and finally are selectively counted in rapid sequence at the detector, allowing individual isotopes of an element to be determined. [9]

Iodine, an essential element for thyroid function, is necessary for normal growth, development, and functioning of the brain and body. Iodine-deficiency disorder (IDD) is a well-documented global health problem that affects more than a billion people worldwide. The consequences of IDD include goiter, cretinism, intellectual impairment, brain damage, mental retardation, stillbirth, congenital deformities, and increased perinatal mortality. The detection methods and procedures for iodine are consistent with those for metals. [9]

Nicotine metabolites: "Total" urinary nicotine metabolites, including the free and glucuronide conjugated forms, are measured by two separate isotope dilution high-performance liquid chromatography/tandem mass spectrometry (HPLC-MS/MS) methods based on a cotinine cut-off value of 20 ng/mL so that markedly different levels of nicotine exposure biomarkers can be optimally quantified in both users and non-users.[10,11]

Perchlorate, nitrate, and thiocyanate: This study outlines a quantitative method for measuring nitrate, perchlorate, and thiocyanate in human urine using ion chromatography coupled with electrospray tandem mass spectrometry. To achieve chromatographic separation, we utilized an IonPac AS16 column with sodium

© 2024 Guo J et al. *JAMA Network Open*.

hydroxide serving as the eluent. Ionization of the eluent in the column was accomplished through an electrospray interface, generating negative ionization and transferring them to the mass spectrometer.[12]

Polycyclic aromatic hydrocarbons (PAHs) constitute a group of chemicals formed during the incomplete combustion of coal, oil and gas, garbage, and other organic substances. In general, people are exposed to mixtures of PAHs, and the sources are widespread, including vehicle exhaust, asphalt, coal tar, wildfires, agricultural burning, charbroiled foods, and tobacco smoke. Upon entering the body, PAHs are readily metabolized and eliminated in urine. The urinary concentrations of PAH metabolites, specifically monohydroxylated PAHs (OH-PAHs), have been used as biomarkers of human exposure to select PAHs including naphthalene, fluorene, phenanthrene, and pyrene. The indicator measured was the monohydroxy metabolite of PAHs (OH-PAHs), namely 1-hydroxynaphthalene, 2-hydroxynaphthalene, 2-hydroxyfluorene, 3-hydroxyfluorene, 1-hydroxyphenanthrene, 2- & 3-hydroxyphenanthrene, and 1-hydroxypyrene. The analytical procedure involves enzymatic hydrolysis of glucuronidated/sulfated OH-PAH metabolites in urine, extraction by on-line solid phase extraction, and separation and quantification using isotope dilution high performance liquid chromatography-tandem mass spectrometry (on-line SPE-HPLC-MS/MS).[13]

Volatile nitrosamines (VNAs) are harmful ingredients into tobacco smoke. It has been reported that VNA exposure may play an important role in the etiology of esophageal cancer and schistosomiasis-associated bladder cancer. Studies have also shown correlations between VNA exposure and lipid peroxidation and oxidative stress (insulin-resistance and inflammation), chronic diseases (diabetes), and neurodegenerative diseases, such as Alzheimer's disease. VNAs in human urine are measured using an isotope dilution gas chromatography tandem mass spectrometric (GC/MS-MS) method.[14]

Volatile organic compounds: Exposure to volatile organic compounds (VOCs) is

© 2024 Guo J et al. *JAMA Network Open*.

ubiquitous. Chronic exposure to extremely high levels of some VOCs can lead to cancer and neurocognitive dysfunction. Urinary metabolites of VOCs can be detected in urine for a longer period of time than the parent VOCs can be detected in blood. Quantification of volatile organic compound metabolites in human urine using ultra performance liquid chromatography-electrospray tandem mass spectrometry (UPLC-ESI/MS/MS).[15]

3. Selection of environmental toxicants to be included in the analyses

We categorized exposure using 3 approaches based on the toxicant detection limit and used values listed below the limit to maximize the available information: 1) a toxicant was not included in the analysis if more than 90% of participants had values below its detection limit or if more than 40% were missing; 2) a toxicant was converted to a binary variable if more than 30% of participants had a value below its detection limit, with these values set to zero;[16] and 3) in all other cases, the variable was natural logarithmically transformed.[16] We imputed missing values for all variables, including all covariates, exposure variables, and mediating variables, using the chained equation method.

4. Exposome-wide association study (ExWAS)

The ExWAS an approach that is widely used in exposome studies, relies on a linear regression that is fitted independently for each covariate.[17-20] After applying multiple comparisons, the association between the predictor and the response variable was evaluated based on the corresponding two-sided p-value. We employed the Benjamini–Hochberg (BH) approach to compute the false discovery rate (FDR) and evaluate the resilience of the discoveries to account for multiple testing.[21,22] When analyzing data of a complex survey, sampling weights are usually used to produce representative and unbiased statistics, which will help to deal with the clustering within household and neighborhoods. However, it reduces the precision of the estimates, and even to some extent introduces over-adjustment bias if variables (e.g. sex, race, age and income) that were used to calculate the sampling weights were further adjusted in regression

© 2024 Guo J et al. *JAMA Network Open*.

analyses.[23,24] Thus, we presented our results without incorporating sampling weights, similar to some previous studies using NHANES data.[25-28]

5. Deletion/Substitution/Addition (DSA) algorithm

DSA is an iterative algorithm for searching linear regression models.[29] Three user-specified constraints limit the set of potential models, including the maximum order of interactions between predictors, the maximum power of a given predictor, and the maximum model size. In each iteration, the following measures were allowed: removing a term, replacing a term with another term, and adding a term to the current model. Using 5-fold cross-validation data, we selected the final model by minimizing the RMSE value. The search for this model began with the intercept model. We have not yet taken into account any polynomials or interaction terms in our model, but we have included all covariates and environmental toxicant variables. Some of the exposome variables were redundant and displayed high correlations that were likely to hinder any multi-exposure regression model. Hence, in DSA, we selected one exposure with high a priori plausibility per exposure indicator and ensured that no pair of exposures displayed an absolute correlation coefficient above 90% (when this occurred, we selected the exposure variable with the smallest proportion of imputed values and removed the other).[30]

6. Mediation analysis framework

To explore the mediating effects of the total peripheral white blood cell (WBC) count, which we use as a surrogate for systemic inflammation, we performed a two-stage mediating effect analysis. Initially, a pairwise mediation analysis was undertaken with outcome Y and all feasible combinations of analytes from exposure matrix X (n_1= 50, where n_1 equals the number of environmental toxicants, n=3,427, where n denotes the number of participants) and total WBC from mediator matrix M (n_2=1, where n_2 is the number of mediators). This results in a total of 300 unique mediation models. We meticulously screened all possible mediator pathways linking each toxicant and inflammatory biomarker assessed in present study. The specific equations of the

mediator model are as follows:

$$M_i = \alpha_\alpha X_i + \alpha_c C_i^T + \epsilon_i \quad (1)$$

In these models, C_i^T, is a covariate vector of *r*-by-*1* ($r = 11$, where the first element is scalar 1 for the intercept and the remainder of the elements are covariates) for the *i*-th participants. In models where depressive symptoms was the binary outcome variable, the model specification was as follow:

$$logit[P(Y_i = 1)|X_i, M_i, C_i^T] = \beta_\alpha X_i + \beta_m M_i + \beta_c C_i^T + \delta_i \quad (2)$$

The equation used to calculate the size of the indirect effect for each toxicant-mediator was: indirect effect=$\alpha_\alpha \times \beta_m$. Additionally, to calculate the confidence interval of the indirect effect, the bootstrap method was employed (1,000 iterations for this study).[31] We also calculated the false discovery rate (FDR) to adjust the p-value of the indirect effect.

Exposure dimension reduction followed by pairwise mediation analysis is another approach to mediation analysis that aims to reduce the dimensionality of exposures to reduce the number of mediating role models. The approach of using exposure-class risk scores (ERS) for mediation analysis is the second option. We formed ERS according to environmental toxicants by class, reducing the size of the exposure matrix from 50 to 5. The purpose of this approach is to reduce bias caused by covariance and to mediate the exposure of environmental toxicants in their respective class k. These include nicotine metabolites (n_1= 3), metals (n_2= 15), perchlorate, nitrate, and thiocyanate (n_3= 3), PAHs (n_4= 7), and VOC metabolites (n_5= 16). The goal is to estimate the cumulative effect of these toxicants. We excluded the other four substances from the calculation of ERS, as they were either too few or unsuitable. Additionally, we did not consider categorical variables. For this reason, we limited the total number of pairwise mediation models to 5. To construct the ERS, we first used adaptive elastic net regularization with elastic networks to estimate the weights of the toxicants.

$$\widehat{\gamma_k} = \underset{\gamma_k}{\arg min} \left\{ \frac{1}{n} \sum_{i=1}^{n} (Y_i - \gamma_c C_i^T - \gamma_k X_{ik}^T)^2 + \lambda_1 \sum_{j=1}^{p_k} |\gamma_{kj}| + \frac{\lambda_2}{2} \sum_{j=1}^{p_k} \gamma_{kj}^2 \right\} \quad (3)$$

© 2024 Guo J et al. *JAMA Network Open*.

The adaptive elasticity network is a combination of minimum absolute contraction and selection operator penalties with ridge regression.[32] In this equation, Y_i is the dichotomous variable. There are p_k environmental toxicants in the k-th exposure class, $X_{ik}^T p_k\, i(i = 1, \dots, n) = 1, \dots, p_k)$, and λ_2 , a tuning parameter, which was used to stabilize the solution path to avoid multicollinearity in the exposure matrix. Prediction error optimization and 5-fold cross-validation were used to estimate λ_1 and λ_2.

To create the ERS for each exposure class, we begin by extracting γ_k, a $1 - by - p_k$ vector of coefficients obtained through an adaptive elastic net, with its elements represented as γkj. These coefficients are designated as weights. Next, we calculate the individual scores (ERS_{ik}) for the k-th exposure class by computing a linear combination. This is achieved by applying the weights derived from the adaptive elastic net across each individual's observed measurements of various toxicants: $ERS_{ik} = \widehat{\gamma_k} X_{ik}^T$. We used the calculated ESR in the first approach to explore the mediating role of inflammatory indicators in the association of different exposure families with depressive symptoms.

7. Covariates

We included covariates in ExWAS, DSA algorithm and mediation analysis framework based on previous research, including sex (categorized into male vs. female), age (categorized into <65 years vs. ≥65 years), race/Hispanic origin (categorized into Mexican American, Non-Hispanic Black, Non-Hispanic White, Other Hispanic, Other Race), family income-to-poverty ratio (converted to a categorical variable according to median [2.06] and interquartile range (IQR, 1.07-3.93)), body mass index (categorized into <25 kg/m^3, 25~30 kg/m^3 and ≥30 kg/m^3), alcohol consumption (had at least 12 drinks [a drink meaning 12 oz. beer, a 5 oz. glass of wine, or one and half ounces of liquor.] of any type of alcoholic beverage, categorized into yes and no) of any type of alcoholic beverage, categorized into yes and no), urine or blood sample collection time (categorized into morning, afternoon and evening), six-month survey period (categorized into November 1 to April 30 and May 1 to October 31), survey cycles

© 2024 Guo J et al. *JAMA Network Open*.

(categorized into 2013~2014 wave and 2015~2016 wave), and sleep duration (categorized into 6-8 h, <6 h, >8 h). Moreover, we adjusted for the glomerular filtration rate by CKD-EPI equation[33,34], an indicator of the renal clearance of environmental toxicants.

© 2024 Guo J et al. *JAMA Network Open*.

eTable 1. Characteristics of 89 Environmental Contaminants or Metabolites of Environmental Contaminants

Family	Exposure	Unit	LOD	Under LOD (%)	Missing (%)	Abnormal value (‰)	Median (IQR)	X̅±SD	Matrix	Label	Process
Acrylamide & Glycidamide	Acrylamide	pmol/g Hb	3.90	0.0	8.3	21.6	42.9(31.4)	63.5±62.6	Serum	Acrylamide	log(ln)
	Glycidamide	pmol/g Hb	4.90	0.0	11.8	21.6	38.4(26.4)	49.5±39.7	Serum	Glycidamide	log(ln)
Aromatic Amines	1-Aminonaphthalene	pg/mL	1.29	28.6	52.1	13.7	2.2(8.0)	17.8±40	Urine	1-Aminonaphthalene	removed
	2-Aminonaphthalene	pg/mL	2.79	25.8	51.8	7.6	5.2(8.3)	10.6±19.7	Urine	2-Aminonaphthalene	removed
	4-Aminobiphenyl	pg/mL	1.75	22.7	51.4	2.3	6.1(9.7)	14.1±45.9	Urine	4-Aminobiphenyl	removed
	o-Anisidine	pg/mL	7.02	2.1	55.1	2.9	47.7(61.2)	78.7±169.9	Urine	o-Anisidine	removed
	2,6-Dimethylaniline	pg/mL	15.67	35.3	54.0	0.3	23.7(42.7)	9528.9±342907.4	Urine	2,6-Dimethylaniline	removed
	o-Toluidine	pg/mL	111.22	15.3	53.0	9.3	345(490)	595.4±963.9	Urine	o-Toluidine	removed
Arsenics - Speciated	Arsenous Acid	μg/L	0.12	39.6	0.4	12.8	0.3(0.6)	0.4±0.5	Urine	Arsenous Acid	2 categories
	Arsenobetaine	μg/L	1.16	51.3	0.4	11.4	0.8(4.0)	9.6±39.3	Urine	Arsenobetaine	2 categories
	Arsenocholine	μg/L	0.11	82.2	0.4	3.8	0.1(0)	0.2±0.6	Urine	Arsenocholine	2 categories
	Monomethylarsonic Acid	μg/L	0.2	36.4	0.4	14.0	0.4(0.6)	0.6±0.6	Urine	Monomethylarsonic Acid	2 categories
	Arsenic acid	μg/L	0.79	98.0	0.4	7.6	0.6(0)	0.6±0.2	Urine	Arsenic acid	removed
	Dimethylarsinic Acid	μg/L	1.91	26.9	0.4	18.7	3.3(4.3)	4.9±5.7	Urine	Dimethylarsinic Acid	log(ln)
Nicotine Metabolites	Total Cotinine	ng/mL	0.03	2.3	1.3	25.1	0.4(100.2)	712±1911.2	Urine	Total Cotinine	log(ln)
	Total Hydroxycotinine	ng/mL	0.03	2.0	1.3	19.6	0.8(172.8)	1510.6±4617.3	Urine	Total Hydroxycotinine	log(ln)
	TNE - 2	nmol/mL	0	0.9	1.3	19.8	0(1.6)	11.9±33.8	Urine	TNE - 2	log(ln)
Ethylene Oxide	Ethylene Oxide	pmol/g Hb	8.2	3.0	6.2	22.5	21.1(27.2)	61.4±104.8	Serum	Hemoglobin adducts of EO	log(ln)
Formaldehyde	Formaldehyde	nmol/g HB	0.67	0.0	13.2	12.8	132.0(23.0)	132.5±19.7	Serum	Formaldehyde	log(ln)
Heterocyclic Aromatic	A-α-C	pg/mL	0.62	46.9	49.9	3.5	0.7(3.4)	11.5±43.4	Urine	2-Amino-9H-pyrido[2,3-b]indole	removed

© 2024 Guo J et al. *JAMA Network Open*.

Amines	GLU-P1	pg/mL	0.31	98.0	49.8	5.5	0.2(0)	0.2±0.1	Urine	2-Amino-6-methyldipyrido[1,2-a:3',2'-d]imidazole	removed
	GLU-P2	pg/mL	0.83	99.7	49.8	1.5	0.6(0)	0.6±0	Urine	2-Aminodipyrido[1,2-a:3',2'-d] imidazole	removed
	Harman	pg/mL	4.59	0.1	49.8	1.5	121(184.8)	272.1±1050.5	Urine	Harman	removed
	IQ	pg/mL	0.37	98.1	49.8	3.2	0.3(0)	0.3±0.3	Urine	2-Amino-3-methyl-3H-imidazo[4,5-f]quinoline	removed
	MeA-α-C	pg/mL	0.33	80.9	49.8	3.8	0.2(0)	0.8±2.9	Urine	2-Amino-3-methyl-9H-pyriodo[2,3-b]indole	removed
	Norharman	pg/mL	12.6	0.1	50.3	8.5	326(442)	501.7±575.4	Urine	Norharman	removed
	Ph1P	pg/mL	0.34	41.5	49.8	8.8	0.5(1.9)	4.3±15.6	Urine	2-Amino-1-methyl-6-phenylimidazo[4,5-b]pyridine	removed
	Trp-P-1	pg/mL	0.79	98.5	49.8	4.1	0.6(0)	0.6±0.3	Urine	3-Amino-1,4-dimethyl-5H-pyrido[4,3-b]indole	removed
	Trp-P-2	pg/mL	0.63	97.7	49.8	5.3	0.5(0)	0.5±0.2	Urine	1-Methyl-3-amino-5H-pyrido[4,3-b]indole	removed
Iodine	Iodine	ng/mL	2.4	0.0	0.1	3.2	122.7(157.4)	296.3±2185.7	Urine	Iodine	log(ln)
Metals	Mercury	ng/mL	0.13	37.9	0.1	9.3	0.2(0.4)	0.4±1	Urine	Inorganic	2 categories
	Copper	µg/L	2.5	0.0	4.3	16.0	115.1(34.6)	119.6±30.5	Serum	Copper	log(ln)
	Selenium	µg/L	4.5	0.0	4.4	8.2	128.8(20.9)	130.1±17.6	Serum	Selenium	log(ln)
	Zinc	µg/L	2.9	0.0	4.4	8.5	80.4(19.5)	81.3±15.2	Serum	Zinc	log(ln)
	Manganese	µg/L	0.13	71.6	0.0	4.7	0.1(0)	0.2±0.6	Urine	Manganese	2 categories
	Barium	µg/L	0.06	0.4	0.0	12.3	1(1.5)	1.7±3	Urine	Barium	log(ln)
	Cadmium	µg/L	0.036	7.8	0.0	20.7	0.2(0.3)	0.3±0.4	Urine	Cadmium	log(ln)
	Cobalt	µg/L	0.023	0.1	0.0	9.3	0.4(0.4)	0.6±1	Urine	Cobalt	log(ln)
	Cesium	µg/L	0.3	0.0	0.0	10.2	4.2(3.9)	4.9±3.8	Urine	Cesium	log(ln)
	Molybdenum	µg/L	0.8	0.0	0.0	16.0	36.8(46.5)	49.7±49.8	Urine	Molybdenum	log(ln)
	Lead	µg/L	0.03	0.6	0.0	10.8	0.3(0.4)	0.5±0.7	Urine	Lead	log(ln)

© 2024 Guo J et al. *JAMA Network Open*.

	Antimony	µg/L	0.022	22.8	0.0	13.1	0(0.1)	0.1±0.1	Urine	Antimony	log(ln)
	Tin	µg/L	0.09	8.5	0.0	12.5	0.4(0.8)	1.2±4.1	Urine	Tin	log(ln)
	Strontium	µg/L	2.34	0.0	0.0	5.8	90.6(104.6)	121±170	Urine	Strontium	log(ln)
	Thallium	µg/L	0.018	0.6	0.0	5.8	0.2(0.2)	0.2±0.2	Urine	Thallium	log(ln)
	Tungsten	µg/L	0.018	17.5	0.0	11.4	0.1(0.1)	0.1±0.2	Urine	Tungsten	log(ln)
	Uranium	µg/L	0.002	18.2	0.0	11.7	0(0)	0±0	Urine	Uranium	log(ln)
Polycyclic Aromatic Hydrocarbons	1-napthol	ng/L	60	0.0	3.0	2.9	1378(3833)	38528.7±772368.7	Urine	1-Hydroxynapthalene	log(ln)
	2-napthol	ng/L	90	0.0	1.7	11.7	5419.5(9447)	9797.6±15896.6	Urine	2-Hydroxynapthalene	log(ln)
	3-hydroxyfluorene	ng/L	8	0.0	0.2	17.8	74(175)	272.5±611.9	Urine	3-Hydroxyfluorene	log(ln)
	2-hydroxyfluorene	ng/L	8	0.0	0.1	16.3	181.5(338.3)	477±988.8	Urine	2-Hydroxyfluorene	log(ln)
	1-hydroxyphenanthrene	ng/L	9	0.0	0.1	11.1	100(134)	166.9±295.3	Urine	1-Hydroxyphenanthrene	log(ln)
	1-hydroxypyrene	ng/L	70	29.0	0.1	10.8	114(171.8)	215.5±431	Urine	1-Hydroxypyrene	log(ln)
	2&3-Hydroxyphenanthrene	ng/L	10	0.0	0.1	10.8	124(167)	222.2±443.1	Urine	2-Hydroxyphenanthrene & 3-Hydroxyphenanthrene	log(ln)
Perchlorate, Nitrate & Thiocyanate	Perchlorate	ng/mL	0.05	0.0	0.0	4.7	2.4(2.9)	3.9±11.3	Urine	Perchlorate	log(ln)
	Nitrate	ng/mL	700	0.0	0.0	12.8	43600(44200)	54988.4±47925	Urine	Nitrate	log(ln)
	Thiocyanate	ng/mL	20	0.1	0.3	16.6	975.5(1771)	2213.3±4183.7	Urine	Thiocyanate	log(ln)
Volatile N-nitrosamine compounds	NDEA	ng/L	5.02	99.6	51.9	1.2	3.6(0)	3.6±1.9	Urine	N-Nitrosodiethylamine	removed
	NMEA	ng/L	3.64	99.5	51.2	1.2	2.6(0)	2.6±0.8	Urine	N-Nitrosoethylmethylamine	removed
	NMQR	ng/L	7.84	94.9	53.6	4.4	5.5(0)	6±4.1	Urine	N 颅 Nitrosomorpholine	removed
	NPIP	ng/L	5.08	95.6	50.7	3.5	3.6(0)	4.6±11.5	Urine	N-Nitrosopiperidine	removed
	NPYR	ng/L	8.15	99.6	49.6	1.5	5.8(0)	5.8±1.7	Urine	N-Nitrosopyrrolidine	removed
Volatile organic compound Metabolites	2,2DCVMA	µg/L	4.7	51.8	4.7	0.0	3.3(6.5)	6.4±3.2	Urine	N-Acetyl-S-(2,2-dichlorovinyl)-L-cysteine	2 categories
	GAMA	µg/L	9.4	58.9	0.2	19.8	6.7(7.8)	13.1±14.1	Urine	N-Acetyl-S-(2-carbamoyl-2-hydroxyethyl)-L-cysteine	2 categories

© 2024 Guo J et al. *JAMA Network Open*.

HEMA	μg/L	0.791	54.0	0.8	18.7	0.6(1)	1.7±2.8	Urine	N-Acetyl-S-(2-hydroxyethyl)-L-cysteine	2 categories
MHBMA2	μg/L	0.7	86.2	0.4	10.8	0.5(0)	0.9±2.2	Urine	N-Acetyl-S-(2-hydroxy-3-butenyl)-L-cysteine	2 categories
PHEMA	μg/L	0.7	55.6	2.5	9.3	0.5(0.8)	1.2±2.2	Urine	N-Acetyl-S-(phenyl-2-hydroxyethyl)-L-cysteine	2 categories
PMA	μg/L	0.6	58.3	1.7	16.6	0.4(0.6)	0.9±1.1	Urine	N-Acetyl-S-(phenyl)-L-cysteine	2 categories
1,2DCVMA	μg/L	12.6	100.0	5.6	0.3	8.9(0)	8.9±0.1	Urine	N-Acetyl-S-(1,2-dichlorovinyl)-L-cysteine	removed
DPMA	μg/L	0.5	99.7	3.0	2.0	0.4(0)	0.4±0.1	Urine	N-Acetyl-S-(dimethylphenyl)-L-cysteine	removed
MHBMA1	μg/L	0.7	99.3	1.5	6.7	0.5(0)	0.5±0	Urine	N-Acetyl-S-(1-hydroxymethyl-2-propenyl)-L-cysteine	removed
TCVMA	μg/L	3	100.0	0.6	0.0	2.1(0)	2.1±0	Urine	N-Acetyl-S-(trichlorovinyl)-L-cysteine	removed
TTCA	μg/L	11.2	0.0	48.9	10.8	7.9(10.9)	29.3±68.8	Urine	2-Thioxothiazolidine-4-carboxylic acid	removed
2MHA	μg/L	5	8.1	5.5	14.0	30.1(61.9)	63.6±110.6	Urine	2-Methylhippuric acid	log(ln)
3&4 MHA	μg/L	8	0.5	0.4	11.1	197(428.9)	449.5±943.6	Urine	3- and 4-Methylhippuric acid	log(ln)
AAMA	μg/L	2.2	0.2	2.8	15.2	50.8(77.1)	88±132.1	Urine	N-Acetyl-S-(2-carbamoylethyl)-L-cysteine	log(ln)
AMCC	μg/L	6.26	0.2	1.2	23.6	136(217.7)	238.2±311.8	Urine	N-Acetyl-S-(N-methylcarbamoyl)-L-cysteine	log(ln)
ATCA	μg/L	15	4.7	2.5	21.0	109(173.5)	175.4±199.3	Urine	2-Aminothiazoline-4-carboxylic acid	log(ln)
BMA	μg/L	0.5	0.8	0.5	10.2	6.5(9.1)	13±33.8	Urine	N-Acetyl-S-(benzyl)-L-cysteine	log(ln)
BPMA	μg/L	1.2	24.8	1.3	12.0	3.4(8.1)	11.2±32.8	Urine	N-Acetyl-S-(n-propyl)-L-cysteine	log(ln)
CEMA	μg/L	6.96	0.9	0.7	16.6	99.5(137.3)	157.7±212.4	Urine	N-Acetyl-S-(2-carboxyethyl)-L-cysteine	log(ln)
CYMA	μg/L	0.5	14.5	1.3	20.7	1.7(8.2)	45.2±132.7	Urine	N-Acetyl-S-(2-cyanoethyl)-L-cysteine	log(ln)
DHBMA	μg/L	5.25	0.0	5.2	13.1	306(332)	372.3±284.7	Urine	N-Acetyl-S-(3,4-dihydroxybutyl)-L-cysteine	log(ln)
2HPMA	μg/L	5.3	5.6	1.1	9.3	28.8(41.6)	68.9±254.5	Urine	N-Acetyl-S-(2-hydroxypropyl)-L-cysteine	log(ln)
3HPMA	μg/L	13	0.2	3.7	14.0	245(381)	484.6±881	Urine	N-Acetyl-S-(3-hydroxypropyl)-L-cysteine	log(ln)

© 2024 Guo J et al. *JAMA Network Open*.

MA	μg/L	12	0.0	1.9	18.4	133(158.3)	188.8±207.3	Urine	Mandelic acid	log(ln)
MHBMA3	μg/L	0.6	3.4	2.5	19.3	4.9(8.1)	11.9±22	Urine	N-Acetyl-S-(4-hydroxy-2-butenyl)-L-cysteine	log(ln)
PGA	μg/L	12	0.0	1.6	16.6	200(240)	267.3±257.6	Urine	Phenylglyoxylic acid	log(ln)
HPMMA	μg/L	1.7	0.0	1.3	19.8	239(338)	497±864.6	Urine	N-Acetyl-S-(3-hydroxypropyl-1-methyl)-L-cysteine	log(ln)

Abbreviations: LOD, limit of detection; IQR, interquartile range; SD, standard deviation.

© 2024 Guo J et al. *JAMA Network Open*.

eTable 2. Characterization of Participants in This Study Before Data Interpolation

Variables	All (N = 3,427)	No-depression (n = 2,588)	Depression (n=839)
**Sex**			
Male	1692(49.4)	1343(51.9)	349(41.6)
Female	1735(50.6)	1245(48.1)	490(58.4)
**Age (years)**			
<65	2683(78.3)	2037(78.7)	646(77.0)
≥65	744(21.7)	551(21.3)	193(23.0)
**Race/Hispanic origin**			
Mexican American	570(16.6)	440(17.0)	130(15.5)
Non-Hispanic Black	679(19.8)	493(19.0)	186(22.2)
Non-Hispanic White	1314(38.3)	988(38.2)	326(38.9)
Other Hispanic	382(11.1)	284(11.0)	98(11.7)
Other Race	482(14.1)	383(14.8)	99(11.8)
**Education**			
Less than 9th grade	371(10.8)	263(10.2)	108(12.9)
9-11th grade	405(11.8)	266(10.3)	139(16.6)
High school graduate/GED or equivalent	799(23.3)	592(22.9)	207(24.7)
Some college or AA degree	1852(54.0)	1467(56.7)	385(45.9)
**Ratio of family income to poverty**			
<1.07	765(22.3)	516(19.9)	249(29.7)
1.07~2.06	790(23.1)	583(22.5)	207(24.7)
2.06~3.93	777(22.7)	602(23.3)	175(20.9)
≥3.93	785(22.9)	655(25.3)	130(15.5)
Missing	310(9.1)	232(9.0)	78(9.3)
**BMI (kg/m^2)**			
<25	1009(29.4)	773(29.9)	236(28.1)
25~30	1091(31.8)	854(33.0)	237(28.2)
≥30	1301(38.0)	939(36.3)	362(43.1)
**Missing**	26(0.8)	22(0.9)	4(0.5)
**Sleep duration (hours)**			
6~8	2366(69.0)	1867(72.1)	499(59.5)
<6	381(11.1)	230(8.89)	151(18.0)
>8	671(19.6)	485(18.7)	186(22.2)
**Missing**	9(0.3)	6(0.2)	3(0.4)
**Alcohol consumption**			
Yes	2399(70.0)	1800(69.6)	599(71.4)
No	1024(29.9)	786(30.4)	238(28.4)
Missing	4(0.1)	2(0.1)	2(0.2)
**eGFR** (mL/min/1.73 m^2)			
<66.56	815(23.8)	610(23.6)	205(24.4)

© 2024 Guo J et al. *JAMA Network Open.*

66.56~84.02	815(23.8)	639(24.7)	176(21.0)
84.02~100.18	815(23.8)	603(23.3)	212(25.3)
≥100.18	816(23.8)	614(23.7)	202(24.1)
**Missing**	166(4.8)	122(4.7)	44(5.2)
**Sample collection time**			
Morning	1612(47.0)	1243(48.0)	369(44.0)
Afternoon	1254(36.6)	928(35.9)	326(38.9)
Evening	561(16.4)	417(16.1)	144(17.2)
**Six-month survey period**			
November 1 through April 30	1716(50.1)	1288(49.8)	428(51.0)
May 1 through October 31	1711(49.9)	1300(50.2)	411(49.0)
**Survey cycles**			
2013~2014	1754(51.2)	1327(51.3)	427(50.9)
2015~2016	1673(48.8)	1261(48.7)	412(49.1)

The categorical data are presented as absolute numbers (percentages).

© 2024 Guo J et al. *JAMA Network Open*.

eTable 3. Adjusted Association Between Environmental Toxicants (62 Exposures) and Depressive Symptoms in 3427 Adults (ExWAS Analysis) Stratified by Age and Sex

Exposure family	Exposure	Sex				Age (years)			
		Male		Female		< 65		≥65	
		OR (95% CI)	P	OR (95% CI)	P	OR (95% CI)	P	OR (95% CI)	P
Acrylamide & Glycidamide	Acrylamide	1.06 (0.94, 1.20)	0.47	1.22 (1.08, 1.38)	0.009	1.13 (1.03, 1.24)	0.03	1.21 (0.98, 1.49)	0.27
Acrylamide & Glycidamide	Glycidamide	1.03 (0.91, 1.17)	0.75	1.17 (1.03, 1.34)	0.06	1.10 (0.99, 1.21)	0.13	1.07 (0.88, 1.32)	0.69
Arsenics - Speciated	Arsenobetaine	1.08 (0.84, 1.38)	0.71	0.95 (0.75, 1.19)	0.76	0.98 (0.81, 1.18)	0.88	1.11 (0.77, 1.60)	0.72
Arsenics - Speciated	Arsenocholine	0.94 (0.69, 1.29)	0.79	0.88 (0.65, 1.20)	0.56	0.86 (0.67, 1.10)	0.32	1.10 (0.70, 1.72)	0.82
Arsenics - Speciated	Arsenous Acid	0.89 (0.69, 1.16)	0.55	0.91 (0.73, 1.15)	0.57	0.87 (0.71, 1.06)	0.26	1.04 (0.72, 1.50)	0.90
Arsenics - Speciated	Dimethylarsinic Acid	1.04 (0.88, 1.22)	0.77	0.90 (0.77, 1.04)	0.27	0.93 (0.82, 1.05)	0.35	1.09 (0.86, 1.40)	0.69
Arsenics - Speciated	Monomethylarsonic Acid	0.88 (0.67, 1.14)	0.47	1.03 (0.82, 1.29)	0.86	0.88 (0.73, 1.07)	0.32	1.30 (0.90, 1.88)	0.33
Ethylene Oxide	Ethylene Oxide	1.13 (1.02, 1.25)	0.06	1.21 (1.10, 1.34)	0.001	1.16 (1.08, 1.26)	<0.001	1.21 (1.01, 1.44)	0.20
Formaldehyde	Formaldehyde	1.07 (0.94, 1.21)	0.47	0.99 (0.89, 1.11)	0.97	1.04 (0.94, 1.14)	0.56	0.97 (0.80, 1.16)	0.82
Iodine	Iodine	0.98 (0.83, 1.16)	0.87	0.91 (0.77, 1.06)	0.36	0.92 (0.80, 1.05)	0.32	0.97 (0.77, 1.23)	0.90
Metals	Copper	1.13 (0.94, 1.35)	0.35	1.02 (0.87, 1.19)	0.86	1.00 (0.87, 1.14)	0.96	1.24 (0.96, 1.60)	0.27
Metals	Selenium	0.97 (0.83, 1.13)	0.77	0.94 (0.82, 1.07)	0.49	0.98 (0.87, 1.10)	0.74	0.86 (0.70, 1.06)	0.33
Metals	Zinc	0.80 (0.67, 0.95)	0.04	1.03 (0.88, 1.21)	0.84	0.96 (0.84, 1.10)	0.63	0.79 (0.61, 1.03)	0.27
Metals	Barium	1.00 (0.86, 1.16)	0.96	0.88 (0.76, 1.02)	0.17	0.93 (0.82, 1.04)	0.32	0.97 (0.79, 1.20)	0.89
Metals	Cadmium	1.24 (1.02, 1.50)	0.07	1.10 (0.92, 1.31)	0.43	1.19 (1.04, 1.37)	0.04	0.99 (0.70, 1.39)	0.98
Metals	Cobalt	1.04 (0.87, 1.25)	0.77	1.02 (0.89, 1.17)	0.86	1.00 (0.88, 1.14)	0.98	1.07 (0.85, 1.34)	0.72
Metals	Cesium	0.95 (0.81, 1.12)	0.71	0.88 (0.75, 1.03)	0.22	0.94 (0.83, 1.07)	0.46	0.82 (0.65, 1.05)	0.28
Metals	Mercury	0.96 (0.74, 1.24)	0.85	1.07 (0.84, 1.35)	0.72	0.94 (0.77, 1.14)	0.60	1.37 (0.93, 2.03)	0.28
Metals	Manganese	1.12 (0.85, 1.48)	0.55	1.28 (1.02, 1.62)	0.12	1.17 (0.96, 1.43)	0.22	1.38 (0.95, 2.02)	0.27

© 2024 Guo J et al. *JAMA Network Open*.

Metals	Molybdenum	0.98 (0.84, 1.15)	0.87	0.88 (0.76, 1.01)	0.14	0.94 (0.83, 1.05)	0.35	0.88 (0.71, 1.08)	0.40
Metals	Lead	1.02 (0.86, 1.22)	0.86	1.11 (0.94, 1.30)	0.36	1.09 (0.95, 1.24)	0.32	1.00 (0.75, 1.32)	1.00
Metals	Antimony	1.22 (1.05, 1.41)	0.03	1.00 (0.87, 1.14)	0.97	1.09 (0.97, 1.22)	0.22	1.11 (0.88, 1.39)	0.60
Metals	Tin	1.20 (1.02, 1.42)	0.07	1.15 (0.99, 1.34)	0.16	1.16 (1.02, 1.32)	0.05	1.21 (0.96, 1.54)	0.28
Metals	Strontium	1.01 (0.88, 1.17)	0.87	0.97 (0.85, 1.11)	0.79	1.01 (0.90, 1.13)	0.88	0.96 (0.79, 1.17)	0.82
Metals	Thallium	0.90 (0.77, 1.06)	0.37	0.87 (0.76, 1.01)	0.14	0.88 (0.78, 1.00)	0.10	0.93 (0.76, 1.15)	0.71
Metals	Tungsten	1.20 (1.03, 1.39)	0.06	0.99 (0.86, 1.15)	0.97	1.07 (0.95, 1.21)	0.35	1.07 (0.85, 1.35)	0.72
Metals	Uranium	1.28 (1.10, 1.50)	0.01	1.08 (0.94, 1.24)	0.43	1.20 (1.06, 1.35)	0.01	1.09 (0.86, 1.38)	0.69
Nicotine Metabolites	TNE-2	1.35 (1.15, 1.59)	0.003	1.48 (1.25, 1.75)	<0.001	1.40 (1.23, 1.59)	<0.001	1.47 (1.10, 1.96)	0.11
Nicotine Metabolites	Total Cotinine	1.28 (1.10, 1.50)	0.01	1.38 (1.18, 1.62)	0.001	1.31 (1.16, 1.48)	<0.001	1.45 (1.11, 1.90)	0.11
Nicotine Metabolites	Total Hydroxycotinine	1.35 (1.15, 1.59)	0.003	1.47 (1.25, 1.74)	<0.001	1.40 (1.23, 1.59)	<0.001	1.46 (1.09, 1.95)	0.11
PAH	1-napthol	1.27 (1.07, 1.49)	0.02	1.32 (1.15, 1.52)	0.001	1.29 (1.15, 1.46)	<0.001	1.30 (1.03, 1.64)	0.19
PAH	2-napthol	1.19 (0.99, 1.43)	0.14	1.26 (1.05, 1.50)	0.06	1.22 (1.05, 1.41)	0.03	1.22 (0.95, 1.58)	0.30
PAH	3-hydroxyfluorene	1.17 (1.00, 1.35)	0.11	1.26 (1.10, 1.46)	0.009	1.22 (1.09, 1.37)	0.002	1.19 (0.93, 1.53)	0.33
PAH	2-hydroxyfluorene	1.27 (1.09, 1.47)	0.01	1.28 (1.11, 1.47)	0.008	1.28 (1.14, 1.43)	<0.001	1.24 (0.97, 1.58)	0.27
PAH	1-hydroxyphenanthrene	1.16 (0.99, 1.36)	0.15	1.11 (0.95, 1.30)	0.31	1.13 (0.99, 1.28)	0.13	1.22 (0.97, 1.53)	0.27
PAH	1-hydroxypyrene	1.23 (1.04, 1.44)	0.04	1.09 (0.92, 1.28)	0.46	1.18 (1.03, 1.34)	0.04	1.08 (0.84, 1.39)	0.72
PAH	2&3-Hydroxyphenanthrene	1.17 (0.99, 1.38)	0.14	1.14 (0.97, 1.33)	0.20	1.18 (1.04, 1.34)	0.03	1.11 (0.87, 1.42)	0.62
Perchlorate, Nitrate & Thiocyanate	Nitrate	1.12 (0.96, 1.31)	0.27	0.99 (0.88, 1.12)	0.97	1.07 (0.95, 1.20)	0.35	1.00 (0.85, 1.18)	0.99
Perchlorate, Nitrate & Thiocyanate	Thiocyanate	1.09 (0.94, 1.26)	0.40	1.12 (0.97, 1.29)	0.22	1.19 (1.06, 1.33)	0.01	0.88 (0.72, 1.08)	0.40
Perchlorate, Nitrate & Thiocyanate	Perchlorate	1.07 (0.91, 1.25)	0.55	0.86 (0.74, 1.00)	0.14	0.97 (0.86, 1.10)	0.73	0.93 (0.74, 1.17)	0.72
VOC Metabolites	2,2DCVMA	1.60 (0.69, 3.69)	0.40	1.20 (0.61, 2.35)	0.72	1.41 (0.79, 2.52)	0.33	1.20 (0.36, 3.98)	0.86
VOC Metabolites	2MHA	1.16 (0.98, 1.38)	0.18	1.13 (0.96, 1.32)	0.24	1.14 (1.00, 1.30)	0.09	1.13 (0.88, 1.45)	0.56
VOC Metabolites	3&4 MHA	1.32 (1.08, 1.62)	0.03	1.19 (0.99, 1.43)	0.14	1.23 (1.06, 1.43)	0.02	1.30 (0.96, 1.75)	0.27
VOC Metabolites	AAMA	1.07 (0.91, 1.25)	0.55	1.18 (1.03, 1.37)	0.08	1.12 (1.00, 1.26)	0.10	1.17 (0.93, 1.49)	0.34

© 2024 Guo J et al. *JAMA Network Open*.

VOC Metabolites	AMCC	1.28 (1.08, 1.52)	0.02	1.09 (0.93, 1.27)	0.43	1.15 (1.01, 1.30)	0.07	1.29 (0.99, 1.68)	0.27
VOC Metabolites	ATCA	1.09 (0.92, 1.29)	0.47	0.85 (0.72, 1.01)	0.14	0.94 (0.82, 1.08)	0.51	0.99 (0.79, 1.26)	0.99
VOC Metabolites	BMA	0.89 (0.77, 1.04)	0.26	0.96 (0.84, 1.09)	0.66	0.95 (0.85, 1.06)	0.46	0.89 (0.71, 1.11)	0.49
VOC Metabolites	BPMA	1.02 (0.86, 1.21)	0.87	0.84 (0.72, 0.98)	0.10	0.96 (0.84, 1.09)	0.62	0.79 (0.61, 1.01)	0.27
VOC Metabolites	CEMA	1.37 (1.17, 1.60)	0.003	1.24 (1.08, 1.42)	0.01	1.24 (1.11, 1.40)	0.001	1.52 (1.21, 1.92)	0.02
VOC Metabolites	CYMA	1.20 (1.09, 1.33)	0.003	1.23 (1.12, 1.36)	0.001	1.20 (1.11, 1.30)	<0.001	1.32 (1.10, 1.58)	0.08
VOC Metabolites	DHBMA	1.20 (1.03, 1.38)	0.04	1.06 (0.93, 1.19)	0.52	1.11 (1.00, 1.23)	0.10	1.16 (0.94, 1.42)	0.33
VOC Metabolites	GAMA	1.26 (0.99, 1.61)	0.14	1.34 (1.07, 1.69)	0.06	1.27 (1.05, 1.53)	0.04	1.51 (1.05, 2.18)	0.18
VOC Metabolites	HEMA	1.32 (1.03, 1.68)	0.07	1.25 (0.99, 1.57)	0.14	1.23 (1.02, 1.48)	0.07	1.55 (1.07, 2.26)	0.17
VOC Metabolites	2HPMA	1.32 (1.14, 1.53)	0.003	1.09 (0.96, 1.24)	0.28	1.21 (1.09, 1.35)	0.002	1.11 (0.89, 1.37)	0.57
VOC Metabolites	3HPMA	1.32 (1.13, 1.54)	0.003	1.15 (1.01, 1.32)	0.13	1.23 (1.10, 1.37)	0.002	1.19 (0.94, 1.50)	0.32
VOC Metabolites	MA	1.21 (1.05, 1.40)	0.04	1.13 (1.00, 1.28)	0.14	1.12 (1.01, 1.25)	0.08	1.36 (1.10, 1.68)	0.10
VOC Metabolites	MHBMA2	1.79 (1.30, 2.46)	0.003	1.68 (1.20, 2.35)	0.01	1.73 (1.35, 2.21)	<0.001	1.86 (0.96, 3.62)	0.27
VOC Metabolites	MHBMA3	1.27 (1.11, 1.46)	0.003	1.18 (1.04, 1.35)	0.06	1.22 (1.11, 1.35)	<0.001	1.23 (0.98, 1.56)	0.27
VOC Metabolites	PHEMA	1.17 (0.91, 1.50)	0.37	1.26 (1.00, 1.58)	0.14	1.19 (0.98, 1.44)	0.13	1.31 (0.91, 1.90)	0.33
VOC Metabolites	PGA	1.10 (0.96, 1.26)	0.30	1.06 (0.94, 1.20)	0.47	1.09 (0.98, 1.21)	0.19	1.04 (0.86, 1.26)	0.82
VOC Metabolites	PMA	0.94 (0.73, 1.20)	0.75	0.99 (0.79, 1.25)	0.97	1.01 (0.83, 1.22)	0.96	0.85 (0.59, 1.24)	0.62
VOC Metabolites	HPMMA	1.31 (1.15, 1.50)	0.003	1.14 (1.01, 1.29)	0.12	1.20 (1.09, 1.33)	0.001	1.33 (1.07, 1.66)	0.11

Abbreviations: 2,2DCVMA, N-acetyl-S-(2,2-dichlorovinyl)-L-cysteine; AAMA, 2-carbamoylethylmercapturic acid; AMCC, methylcarbamoylmercapturate; ATCA, 2-aminothiazoline-4-carboxylic acid; BMA, benzylmercapturic acid; BPMA, N-acetyl-S-(n-propyl)-L-cysteine; CEMA, N-acetyl-S-(2-carboxyethyl)-L-cysteine; CYMA, N-acetyl-S-(2-cyanoethyl)-L-cysteine; DHBMA, dihydroxy-butyl-mercapturic acid; ExWAS, exposome-wide association study; GAMA, carbamoyl-2-hydroxyethylmercapturate; HEMA, 2-hydroxyethylmercapturic acid; HPMA, hydroxypropylmercapturic acid; HPMMA, 3-hydroxy-1-methyl-propylmercapturic acid; MA, mandelic acid; MHA, methylhippuric acid; MHBMA, N-acetyl-S-(2-hydroxy-3-butenyl)-L-cysteine; OR, odds ratio; CI, confidence interval; PAH, polycyclic aromatic hydrocarbon; PGA, polyglutamic acid; PHEMA, poly(2-hydroxyethyl methacrylate); PMA, phenylmercapturic acid; TNE-2,

© 2024 Guo J et al. *JAMA Network Open*.

total nicotine equivalent-2; VOC, volatile organic compound.
ExWAS analyses were performed, adjusting for sex, age, race/Hispanic origin, education, ratio of family income to poverty, BMI, sleep duration, alcohol consumption, estimated glomerular filtration rate, sample collection time, six-month survey period, and survey cycles.
The p value was adjusted to control the false discovery rate at 5%.

© 2024 Guo J et al. *JAMA Network Open*.

eTable 4. Adjusted Association Between Environmental Toxicants (62 Exposures) and Depressive Symptoms in 3427 Adults (ExWAS Analysis), Compared With Threshold-Based Results

Exposure family	Exposure	Processing	IQR	Cutoff at 9 scores		Cutoff at 14 scores	
				**OR (95% CI)**	**P-value**	**OR (95% CI)**	**P-value**
Acrylamide & Glycidamide	Acrylamide	log(ln)	31.4	1.21 (1.06, 1.37)	0.02	1.09 (0.91, 1.32)	0.52
	Glycidamide	log(ln)	26.4	1.18 (1.03, 1.35)	0.04	1.11 (0.91, 1.35)	0.49
Arsenics - Speciated	Arsenobetaine	2 categories	NA	1.04 (0.80, 1.35)	0.83	0.97 (0.66, 1.42)	0.86
	Arsenocholine	2 categories	NA	0.55 (0.36, 0.82)	0.01	0.55 (0.30, 1.03)	0.17
	Arsenous Acid	2 categories	NA	0.78 (0.60, 1.02)	0.14	0.78 (0.53, 1.17)	0.44
	Dimethylarsinic Acid	log(ln)	4.3	0.86 (0.72, 1.03)	0.16	0.79 (0.60, 1.03)	0.21
	Monomethylarsonic Acid	2 categories	NA	1.07 (0.82, 1.40)	0.74	1.16 (0.78, 1.72)	0.59
Ethylene Oxide	Ethylene Oxide	log(ln)	27.2	1.21 (1.10, 1.34)	0.003	1.20 (1.03, 1.39)	0.11
Formaldehyde	Formaldehyde	log(ln)	23.0	1.12 (0.98, 1.29)	0.16	1.22 (0.99, 1.50)	0.18
Iodine	Iodine	log(ln)	157.4	0.95 (0.79, 1.14)	0.71	0.91 (0.69, 1.19)	0.6
Metals	Copper	log(ln)	34.6	1.10 (0.91, 1.32)	0.44	1.48 (1.12, 1.96)	0.04
	Selenium	log(ln)	20.9	1.01 (0.86, 1.19)	0.92	0.92 (0.73, 1.17)	0.60
	Zinc	log(ln)	19.5	0.97 (0.81, 1.17)	0.83	0.89 (0.68, 1.16)	0.56
	Barium	log(ln)	1.5	0.85 (0.72, 0.99)	0.09	0.91 (0.72, 1.15)	0.57
	Cadmium	log(ln)	0.3	0.97 (0.79, 1.18)	0.83	1.05 (0.78, 1.41)	0.81
	Cobalt	log(ln)	0.4	0.93 (0.78, 1.10)	0.52	0.85 (0.66, 1.10)	0.44
	Cesium	log(ln)	3.9	0.82 (0.69, 0.98)	0.07	0.68 (0.53, 0.87)	0.02
	Mercury	2 categories	NA	0.96 (0.73, 1.25)	0.83	0.70 (0.48, 1.04)	0.21
	Manganese	2 categories	NA	1.16 (0.88, 1.53)	0.43	1.18 (0.79, 1.77)	0.57
	Molybdenum	log(ln)	46.5	0.86 (0.74, 1.02)	0.14	0.77 (0.61, 0.98)	0.14

© 2024 Guo J et al. *JAMA Network Open*.

	Lead	log(ln)	0.4	0.95 (0.79, 1.15)	0.73	0.93 (0.71, 1.22)	0.67
	Antimony	log(ln)	0.1	1.00 (0.86, 1.17)	1.00	1.15 (0.92, 1.43)	0.44
	Tin	log(ln)	0.8	1.26 (1.06, 1.49)	0.03	1.24 (0.97, 1.60)	0.22
	Strontium	log(ln)	104.6	0.93 (0.80, 1.07)	0.44	0.92 (0.74, 1.14)	0.59
	Thallium	log(ln)	0.2	0.76 (0.65, 0.90)	0.006	0.53 (0.41, 0.67)	<0.001
	Tungsten	log(ln)	0.1	1.01 (0.85, 1.19)	0.95	0.94 (0.73, 1.20)	0.67
	Uranium	log(ln)	0.0	1.04 (0.87, 1.23)	0.81	1.08 (0.84, 1.39)	0.62
Nicotine Metabolites	TNE-2	log(ln)	1.6	1.59 (1.34, 1.90)	<0.001	1.60 (1.24, 2.06)	0.007
	Total Cotinine	log(ln)	100.2	1.44 (1.22, 1.69)	<0.001	1.48 (1.17, 1.88)	0.02
	Total Hydroxycotinine	log(ln)	172.8	1.60 (1.35, 1.91)	<0.001	1.60 (1.24, 2.06)	0.007
PAH	1-napthol	log(ln)	3833.0	1.36 (1.16, 1.59)	0.003	1.34 (1.07, 1.67)	0.07
	2-napthol	log(ln)	9447.0	1.13 (0.93, 1.38)	0.37	1.15 (0.85, 1.55)	0.54
	3-hydroxyfluorene	log(ln)	175.0	1.15 (0.99, 1.35)	0.14	1.17 (0.94, 1.47)	0.37
	2-hydroxyfluorene	log(ln)	338.3	1.25 (1.07, 1.45)	0.02	1.22 (0.98, 1.53)	0.21
	1-hydroxyphenanthrene	log(ln)	134.0	1.07 (0.90, 1.27)	0.58	1.03 (0.79, 1.33)	0.86
	1-hydroxypyrene	log(ln)	171.8	1.03 (0.86, 1.23)	0.83	0.94 (0.72, 1.23)	0.72
	2&3-Hydroxyphenanthrene	log(ln)	167.0	1.10 (0.93, 1.32)	0.42	1.06 (0.82, 1.37)	0.72
Perchlorate, Nitrate & Thiocyanate	Nitrate	log(ln)	44200.0	0.93 (0.80, 1.07)	0.44	0.84 (0.69, 1.02)	0.22
	Thiocyanate	log(ln)	1771.0	1.09 (0.94, 1.27)	0.41	1.08 (0.86, 1.35)	0.6
	Perchlorate	log(ln)	2.9	0.91 (0.77, 1.08)	0.44	0.87 (0.68, 1.13)	0.49
VOC Metabolites	2,2DCVMA	2 categories	NA	0.79 (0.39, 1.61)	0.67	0.61 (0.24, 1.51)	0.49
	2MHA	log(ln)	61.9	1.21 (1.01, 1.45)	0.09	1.14 (0.87, 1.48)	0.52
	3&4 MHA	log(ln)	428.9	1.45 (1.18, 1.79)	0.003	1.21 (0.89, 1.65)	0.44
	AAMA	log(ln)	77.1	1.20 (1.02, 1.41)	0.07	1.10 (0.86, 1.40)	0.59
	AMCC	log(ln)	217.7	1.30 (1.09, 1.55)	0.02	1.20 (0.92, 1.56)	0.37

© 2024 Guo J et al. *JAMA Network Open*.

	ATCA	log(ln)	173.5	0.83 (0.69, 1.00)	0.09	0.67 (0.52, 0.88)	0.04
	BMA	log(ln)	9.1	0.99 (0.85, 1.16)	0.92	0.88 (0.69, 1.12)	0.49
	BPMA	log(ln)	8.1	0.81 (0.68, 0.98)	0.07	0.63 (0.48, 0.84)	0.02
	CEMA	log(ln)	137.3	1.22 (1.04, 1.43)	0.04	1.30 (1.03, 1.63)	0.13
	CYMA	log(ln)	8.2	1.21 (1.09, 1.34)	0.003	1.23 (1.05, 1.43)	0.07
	DHBMA	log(ln)	332.0	1.20 (1.04, 1.39)	0.04	1.23 (0.99, 1.52)	0.19
	GAMA	2 categories	NA	1.32 (1.02, 1.72)	0.08	1.47 (1.00, 2.15)	0.18
	HEMA	2 categories	NA	1.42 (1.09, 1.85)	0.03	1.24 (0.84, 1.82)	0.49
	2HPMA	log(ln)	41.6	1.13 (0.98, 1.32)	0.17	1.03 (0.82, 1.29)	0.85
	3HPMA	log(ln)	381.0	1.24 (1.06, 1.44)	0.02	1.22 (0.98, 1.52)	0.21
	MA	log(ln)	158.3	1.23 (1.05, 1.42)	0.03	1.26 (1.01, 1.57)	0.17
	MHBMA2	2 categories	NA	1.78 (1.29, 2.46)	0.004	1.88 (1.19, 2.97)	0.06
	MHBMA3	log(ln)	8.1	1.29 (1.12, 1.48)	0.003	1.23 (1.00, 1.50)	0.18
	PHEMA	2 categories	NA	1.41 (1.08, 1.84)	0.03	1.32 (0.90, 1.94)	0.37
	PGA	log(ln)	240.0	1.17 (1.01, 1.36)	0.07	1.09 (0.89, 1.35)	0.56
	PMA	2 categories	NA	1.12 (0.86, 1.46)	0.53	1.15 (0.78, 1.69)	0.60
	HPMMA	log(ln)	338.0	1.28 (1.12, 1.46)	0.003	1.13 (0.92, 1.37)	0.44

Abbreviations: 2,2DCVMA, N-acetyl-S-(2,2-dichlorovinyl)-L-cysteine; AAMA, 2-carbamoylethylmercapturic acid; AMCC, methylcarbamoylmercapturate; ATCA, 2-aminothiazoline-4-carboxylic acid; BMA, benzylmercapturic acid; BPMA, N-acetyl-S-(n-propyl)-L-cysteine; CEMA, N-acetyl-S-(2-carboxyethyl)-L-cysteine; CYMA, N-acetyl-S-(2-cyanoethyl)-L-cysteine; DHBMA, dihydroxy-butyl-mercapturic acid; ExWAS, exposome-wide association study; GAMA, carbamoyl-2-hydroxyethylmercapturate; HEMA, 2-hydroxyethylmercapturic acid; HPMA, hydroxypropylmercapturic acid; HPMMA, 3-hydroxy-1-methyl-propylmercapturic acid; MA, mandelic acid; MHA, methylhippuric acid; MHBMA, N-acetyl-S-(2-hydroxy-3-butenyl)-L-cysteine; NA, not applicable; OR, odds ratio; CI, confidence interval; PAH, polycyclic aromatic hydrocarbon; PGA, polyglutamic acid; PHEMA, poly(2-hydroxyethyl methacrylate); PMA, phenylmercapturic acid; TNE-2, total nicotine equivalent-2; VOC, volatile organic compound.

© 2024 Guo J et al. *JAMA Network Open*.

ExWAS analyses were performed, adjusting for sex, age, race/Hispanic origin, education, the ratio of family income to poverty, BMI, sleep duration, alcohol consumption, estimated glomerular filtration rate, sample collection time, six-month survey period, and survey cycles.
The p value was adjusted to control the false discovery rate at 5%.

© 2024 Guo J et al. *JAMA Network Open*.

eTable 5. Sensitivity Analyses of the Adjusted Associations Between the Environmental Toxicants (62 Exposures) and Depressive Symptoms (ExWAS Analysis)

Exposure family	Exposure	Processing	IQR	Sensitive analysis a		Sensitive analysis b	
				OR (95% CI)	P-value	OR (95% CI)	P-value
Acrylamide & Glycidamide	Acrylamide	log(ln)	31.4	1.18 (1.06, 1.32)	0.006	1.11 (1.03, 1.21)	0.03
	Glycidamide	log(ln)	26.4	1.14 (1.01, 1.27)	0.06	1.07 (0.99, 1.17)	0.17
Arsenics - Speciated	Arsenobetaine	2 categories	NA	1.04 (0.87, 1.25)	0.73	1.00 (0.85, 1.18)	0.99
	Arsenocholine	2 categories	NA	0.99 (0.78, 1.24)	0.91	0.88 (0.71, 1.10)	0.35
	Arsenous Acid	2 categories	NA	0.95 (0.79, 1.15)	0.68	0.92 (0.78, 1.09)	0.41
	Dimethylarsinic Acid	log(ln)	4.3	0.96 (0.85, 1.08)	0.59	0.96 (0.86, 1.07)	0.54
	Monomethylarsonic Acid	2 categories	NA	0.93 (0.78, 1.12)	0.56	0.96 (0.81, 1.14)	0.69
Ethylene Oxide	Ethylene Oxide	log(ln)	27.2	1.19 (1.09, 1.30)	<0.001	1.15 (1.07, 1.23)	<0.001
Formaldehyde	Formaldehyde	log(ln)	23.0	1.01 (0.90, 1.14)	0.9	1.03 (0.95, 1.11)	0.62
Iodine	Iodine	log(ln)	157.4	0.96 (0.85, 1.09)	0.6	0.93 (0.83, 1.04)	0.31
Metals	Copper	log(ln)	34.6	1.02 (0.89, 1.17)	0.77	1.06 (0.95, 1.19)	0.4
	Selenium	log(ln)	20.9	0.95 (0.85, 1.07)	0.53	0.95 (0.86, 1.04)	0.36
	Zinc	log(ln)	19.5	0.92 (0.81, 1.05)	0.29	0.93 (0.83, 1.04)	0.27
	Barium	log(ln)	1.5	0.93 (0.83, 1.05)	0.31	0.95 (0.86, 1.05)	0.38
	Cadmium	log(ln)	0.3	1.16 (1.01, 1.34)	0.07	1.11 (0.98, 1.25)	0.17
	Cobalt	log(ln)	0.4	1.03 (0.92, 1.17)	0.66	1.02 (0.91, 1.13)	0.81
	Cesium	log(ln)	3.9	0.91 (0.81, 1.03)	0.21	0.90 (0.81, 1.01)	0.12
	Mercury	2 categories	NA	1.04 (0.86, 1.25)	0.73	1.05 (0.88, 1.24)	0.66
	Manganese	2 categories	NA	1.28 (1.06, 1.55)	0.02	1.20 (1.01, 1.43)	0.08
	Molybdenum	log(ln)	46.5	0.93 (0.83, 1.04)	0.29	0.94 (0.85, 1.03)	0.27

© 2024 Guo J et al. *JAMA Network Open*.

	Lead	log(ln)	0.4	1.10 (0.97, 1.25)	0.21	1.04 (0.93, 1.16)	0.62
	Antimony	log(ln)	0.1	1.08 (0.97, 1.20)	0.23	1.08 (0.98, 1.19)	0.19
	Tin	log(ln)	0.8	1.19 (1.05, 1.34)	0.01	1.17 (1.05, 1.30)	0.01
	Strontium	log(ln)	104.6	0.98 (0.88, 1.09)	0.75	0.99 (0.90, 1.09)	0.9
	Thallium	log(ln)	0.2	0.89 (0.79, 0.99)	0.07	0.89 (0.81, 0.99)	0.06
	Tungsten	log(ln)	0.1	1.10 (0.98, 1.23)	0.18	1.08 (0.97, 1.19)	0.23
	Uranium	log(ln)	0.0	1.20 (1.07, 1.35)	0.006	1.17 (1.06, 1.29)	0.01
Nicotine Metabolites	TNE-2	log(ln)	1.6	1.48 (1.29, 1.70)	<0.001	1.35 (1.21, 1.50)	<0.001
	Total Cotinine	log(ln)	100.2	1.40 (1.22, 1.59)	<0.001	1.27 (1.15, 1.41)	<0.001
	Total Hydroxycotinine	log(ln)	172.8	1.51 (1.31, 1.73)	<0.001	1.35 (1.21, 1.50)	<0.001
PAH	1-napthol	log(ln)	3833.0	1.30 (1.15, 1.46)	<0.001	1.26 (1.14, 1.39)	<0.001
	2-napthol	log(ln)	9447.0	1.28 (1.11, 1.47)	0.003	1.20 (1.07, 1.36)	0.01
	3-hydroxyfluorene	log(ln)	175.0	1.24 (1.11, 1.39)	<0.001	1.18 (1.07, 1.30)	0.003
	2-hydroxyfluorene	log(ln)	338.3	1.30 (1.16, 1.46)	<0.001	1.24 (1.12, 1.37)	<0.001
	1-hydroxyphenanthrene	log(ln)	134.0	1.20 (1.06, 1.35)	0.01	1.11 (1.00, 1.24)	0.09
	1-hydroxypyrene	log(ln)	171.8	1.20 (1.06, 1.37)	0.01	1.15 (1.03, 1.28)	0.04
	2&3-Hydroxyphenanthrene	log(ln)	167.0	1.19 (1.05, 1.34)	0.01	1.14 (1.03, 1.27)	0.04
Perchlorate, Nitrate & Thiocyanate	Nitrate	log(ln)	44200.0	1.03 (0.93, 1.14)	0.68	1.04 (0.95, 1.13)	0.5
	Thiocyanate	log(ln)	1771.0	1.07 (0.96, 1.20)	0.29	1.09 (0.99, 1.20)	0.12
	Perchlorate	log(ln)	2.9	0.96 (0.85, 1.07)	0.56	0.96 (0.87, 1.07)	0.56
VOC Metabolites	2,2DCVMA	2 categories	NA	1.07 (0.88, 1.29)	NA	0.98 (0.67, 1.46)	0.95
	2MHA	log(ln)	61.9	1.11 (0.97, 1.26)	0.21	1.13 (1.02, 1.26)	0.06
	3&4 MHA	log(ln)	428.9	1.23 (1.06, 1.42)	0.01	1.23 (1.08, 1.39)	0.008
	AAMA	log(ln)	77.1	1.16 (1.03, 1.31)	0.03	1.13 (1.02, 1.25)	0.04
	AMCC	log(ln)	217.7	1.21 (1.06, 1.37)	0.009	1.15 (1.03, 1.28)	0.04

© 2024 Guo J et al. *JAMA Network Open*.

	ATCA	log(ln)	173.5	0.91 (0.80, 1.05)	0.28	0.97 (0.87, 1.08)	0.66
	BMA	log(ln)	9.1	0.91 (0.81, 1.01)	0.15	0.93 (0.84, 1.02)	0.19
	BPMA	log(ln)	8.1	0.91 (0.80, 1.03)	0.22	0.93 (0.83, 1.04)	0.28
	CEMA	log(ln)	137.3	1.28 (1.14, 1.44)	<0.001	1.28 (1.16, 1.41)	<0.001
	CYMA	log(ln)	8.2	1.26 (1.15, 1.37)	<0.001	1.20 (1.12, 1.28)	<0.001
	DHBMA	log(ln)	332.0	1.13 (1.02, 1.26)	0.05	1.12 (1.02, 1.22)	0.04
	GAMA	2 categories	NA	1.36 (1.13, 1.63)	0.004	1.27 (1.08, 1.50)	0.01
	HEMA	2 categories	NA	1.35 (1.12, 1.62)	0.005	1.22 (1.04, 1.43)	0.04
	2HPMA	log(ln)	41.6	1.19 (1.07, 1.32)	0.004	1.17 (1.07, 1.28)	0.004
	3HPMA	log(ln)	381.0	1.22 (1.09, 1.37)	0.003	1.21 (1.10, 1.33)	<0.001
	MA	log(ln)	158.3	1.18 (1.06, 1.31)	0.006	1.14 (1.04, 1.25)	0.01
	MHBMA2	2 categories	NA	1.72 (1.34, 2.20)	<0.001	1.67 (1.33, 2.09)	<0.001
	MHBMA3	log(ln)	8.1	1.21 (1.09, 1.35)	0.002	1.20 (1.10, 1.31)	<0.001
	PHEMA	2 categories	NA	1.33 (1.11, 1.60)	0.007	1.17 (0.99, 1.38)	0.12
	PGA	log(ln)	240.0	1.09 (0.99, 1.21)	0.15	1.07 (0.98, 1.16)	0.22
	PMA	2 categories	NA	1.07 (0.89, 1.28)	0.59	0.96 (0.81, 1.13)	0.66
	HPMMA	log(ln)	338.0	1.23 (1.11, 1.36)	<0.001	1.20 (1.10, 1.31)	<0.001

Abbreviations: 2,2DCVMA, N-acetyl-S-(2,2-dichlorovinyl)-L-cysteine; AAMA, 2-carbamoylethylmercapturic acid; AMCC, methylcarbamoylmercapturate; ATCA, 2-aminothiazoline-4-carboxylic acid; BMA, benzylmercapturic acid; BPMA, N-acetyl-S-(n-propyl)-L-cysteine; CEMA, N-acetyl-S-(2-carboxyethyl)-L-cysteine; CYMA, N-acetyl-S-(2-cyanoethyl)-L-cysteine; DHBMA, dihydroxy-butyl-mercapturic acid; ExWAS, exposome-wide association study; GAMA, carbamoyl-2-hydroxyethylmercapturate; HEMA, 2-hydroxyethylmercapturic acid; HPMA, hydroxypropylmercapturic acid; HPMMA, 3-hydroxy-1-methyl-propylmercapturic acid; MA, mandelic acid; MHA, methylhippuric acid; MHBMA, N-acetyl-S-(2-hydroxy-3-butenyl)-L-cysteine; NA, not applicable; OR, odds ratio; CI, confidence interval; PAH, polycyclic aromatic hydrocarbon; PGA, polyglutamic acid; PHEMA, poly(2-hydroxyethyl methacrylate); PMA, phenylmercapturic acid; TNE-2, total nicotine equivalent-2; VOC, volatile organic compound.

© 2024 Guo J et al. *JAMA Network Open*.

ExWAS was performed, adjusting for sex, age, race/Hispanic origin, education, the ratio of family income to poverty, BMI, sleep duration, alcohol consumption, estimated glomerular filtration rate, sample collection time, six-month survey period, and survey cycles.
Sensitivity analysis was conducted based on data that did not impute missing values.
Sensitivity analysis b was conducted based on data that did not exclude participants with missing values for more than 1/3 of all environmental toxicants.
The P value was adjusted to control the false discovery rate at 5%.

© 2024 Guo J et al. *JAMA Network Open*.

eTable 6. Mediation of Association Between Environmental Toxicants and Depressive Symptoms by Inflammation Biomarkers

Exposure family	Exposure	Mediators	Indirect effect			Total effect		Direct effect		Proportion of mediation	
			Estimate (95% CI)	P	P*	Estimate (95% CI)	P*	Estimate (95% CI)	P*	Estimate (95% CI)	P*
Acrylamide & Glycidamide	Acrylamide	Total WBC	0.00 (0.00, 0.01)	0.002	0.005	0.01 (0.00, 0.01)	0.04	0.01 (0.00, 0.01)	0.04	0.19 (0.07, 0.62)	0.02
Acrylamide & Glycidamide	Glycidamide	Total WBC	0.01 (0.00, 0.01)	<0.001	<0.001	0.01 (-0.02, 0.01)	0.50	0.01 (-0.02, 0.01)	0.50	0.50 (-3.28, 4.55)	0.24
Arsenics - Speciated	Dimethylarsinic Acid	Total WBC	0.00 (0.00, 0.02)	<0.001	<0.001	0.01 (-0.03, 0.01)	0.28	0.01 (-0.03, 0.01)	0.28	0.27 (-1.00, 1.66)	0.16
Ethylene Oxide	Ethylene Oxide	Total WBC	0.01 (-0.00, 0.02)	0.12	0.16	-0.06 (-0.14, 0.01)	0.50	-0.06 (-0.14, 0.01)	0.50	-0.19 (-2.13, 1.77)	0.71
Formaldehyde	Formaldehyde	Total WBC	-0.00 (-0.01, 0.01)	0.97	0.97	-0.10 (-0.15, 0.01)	0.28	-0.10 (-0.15, 0.01)	0.28	0.00 (-0.44, 0.49)	0.96
Iodine	Iodine	Total WBC	-0.00 (-0.01, 0.00)	0.38	0.42	0.01 (-0.11, 0.01)	0.49	0.01 (-0.11, 0.01)	0.49	-0.08 (-0.64, 0.72)	0.72
Metals	Copper	Total WBC	0.00 (0.00, 0.00)	0.008	0.01	0.01 (0.01, 0.02)	<0.001	0.01 (0.01, 0.02)	<0.001	0.13 (0.04, 0.29)	0.03
Metals	Selenium	Total WBC	-0.00 (-0.00, -0.00)	<0.001	<0.001	-0.01 (-0.01, 0.01)	0.33	-0.01 (-0.01, 0.01)	0.33	0.21 (-1.65, 2.07)	0.24
Metals	Zinc	Total WBC	-0.00 (-0.01, -0.00)	0.05	0.07	-0.03 (-0.07, 0.00)	0.14	-0.03 (-0.07, 0.00)	0.14	0.08 (-0.05, 0.37)	0.17
Metals	Barium	Total WBC	0.00 (0.00, 0.00)	0.002	0.005	0.01 (-0.00, 0.01)	0.27	0.01 (-0.00, 0.01)	0.27	0.26 (-0.34, 2.02)	0.13
Metals	Cadmium	Total WBC	0.00 (0.00, 0.00)	0.006	0.01	0.01 (0.01, 0.02)	<0.001	0.01 (0.01, 0.02)	<0.001	0.08 (0.03, 0.17)	0.03
Metals	Cobalt	Total WBC	0.00 (0.00, 0.00)	0.002	0.005	0.01 (-0.00, 0.01)	0.16	0.01 (-0.00, 0.01)	0.16	0.25 (0.06, 1.09)	0.09
Metals	Cesium	Total WBC	0.00 (0.00, 0.00)	0.002	0.005	0.01 (-0.00, 0.02)	0.14	0.01 (-0.00, 0.02)	0.14	0.23 (0.05, 0.91)	0.09
Metals	Molybdenum	Total WBC	0.00 (0.00, 0.01)	0.004	0.008	0.02 (0.00, 0.03)	0.02	0.02 (0.00, 0.03)	0.02	0.19 (0.05, 0.51)	0.02
Metals	Lead	Total WBC	0.00 (0.00, 0.01)	<0.001	<0.001	0.01 (-0.01, 0.03)	0.33	0.01 (-0.01, 0.03)	0.33	0.25 (-1.26, 1.76)	0.23
Metals	Antimony	Total WBC	0.00 (0.00, 0.00)	<0.001	<0.001	0.00 (-0.01, 0.02)	0.56	0.00 (-0.01, 0.02)	0.56	0.37 (-3.56, 2.44)	0.47
Metals	Tin	Total WBC	0.00 (0.00, 0.01)	<0.001	<0.001	0.01 (-0.01, 0.03)	0.29	0.01 (-0.01, 0.03)	0.29	0.26 (-1.05, 1.63)	0.16
Metals	Strontium	Total WBC	-0.00 (-0.00, -0.00)	0.05	0.07	-0.01 (-0.01, 0.00)	0.13	-0.01 (-0.01, 0.00)	0.13	0.07 (-0.04, 0.42)	0.16
Metals	Thallium	Total WBC	-0.00 (-0.00, 0.00)	0.92	0.94	-0.01 (-0.02, 0.01)	0.48	-0.01 (-0.02, 0.01)	0.48	0.01 (-0.74, 0.72)	0.95
Metals	Tungsten	Total WBC	0.00 (-0.00, 0.00)	0.25	0.30	-0.01 (-0.01, -0.01)	0.007	-0.01 (-0.01, -0.01)	0.007	-0.02 (-0.11, 0.02)	0.36
Metals	Uranium	Total WBC	0.00 (-0.00, 0.00)	0.66	0.69	0.01 (-0.01, 0.04)	0.43	0.01 (-0.01, 0.04)	0.43	0.03 (-0.36, 0.77)	0.78

© 2024 Guo J et al. *JAMA Network Open*.

Nicotine Metabolites	Total Cotinine	Total WBC	0.00 (-0.00, 0.00)	0.36	0.42	-0.01 (-0.01, 0.01)	0.32	-0.01 (-0.01, 0.01)	0.32	-0.06 (-0.87, 0.87)	0.63
Nicotine Metabolites	Total Hydroxycotinine	Total WBC	-0.00 (-0.00, 0.00)	0.06	0.09	-0.01 (-0.01, -0.00)	0.07	-0.01 (-0.01, -0.00)	0.07	0.06 (-0.01, 0.26)	0.16
Nicotine Metabolites	TNE-2	Total WBC	-0.00 (-0.00, 0.00)	0.37	0.42	-0.01 (-0.02, 0.01)	0.49	-0.01 (-0.02, 0.01)	0.49	0.08 (-0.68, 0.85)	0.68
PAH	1-napthol	Total WBC	-0.00 (-0.00, 0.00)	0.39	0.43	-0.01 (-0.01, 0.01)	0.26	-0.01 (-0.01, 0.01)	0.26	0.04 (-0.26, 0.46)	0.58
PAH	2-napthol	Total WBC	0.00 (-0.00, 0.00)	0.40	0.43	0.00 (-0.01, 0.04)	0.86	0.00 (-0.01, 0.04)	0.86	0.29 (-0.77, 0.69)	0.94
PAH	3-hydroxyfluorene	Total WBC	0.00 (-0.00, 0.00)	0.25	0.30	0.02 (0.00, 0.05)	0.01	0.02 (0.00, 0.05)	0.01	0.03 (-0.02, 0.13)	0.35
PAH	2-hydroxyfluorene	Total WBC	0.00 (-0.00, 0.00)	0.13	0.17	-0.02 (-0.03, -0.00)	0.07	-0.02 (-0.03, -0.00)	0.07	-0.05 (-0.34, 0.03)	0.24
PAH	1-hydroxyphenanthrene	Total WBC	-0.00 (-0.00, -0.00)	0.02	0.03	-0.00 (-0.01, -0.00)	0.007	-0.00 (-0.01, -0.00)	0.007	0.05 (0.01, 0.15)	0.05
PAH	1-hydroxypyrene	Total WBC	0.00 (-0.00, 0.00)	0.20	0.25	0.01 (-0.00, 0.04)	0.32	0.01 (-0.00, 0.04)	0.32	0.07 (-0.58, 0.62)	0.47
PAH	2&3-Hydroxyphenanthrene	Total WBC	0.00 (0.00, 0.00)	0.02	0.03	0.01 (-0.00, 0.03)	0.50	0.01 (-0.00, 0.03)	0.50	0.19 (-1.67, 1.41)	0.47
Perchlorate, Nitrate & Thiocyanate	Perchlorate	Total WBC	0.00 (0.00, 0.00)	<0.001	<0.001	0.01 (-0.00, 0.02)	0.26	0.01 (-0.00, 0.02)	0.26	0.20 (-0.39, 1.55)	0.13
Perchlorate, Nitrate & Thiocyanate	Nitrate	Total WBC	0.00 (0.00, 0.00)	0.002	0.005	0.02 (0.01, 0.03)	0.007	0.02 (0.01, 0.03)	0.007	0.10 (0.03, 0.30)	0.02
Perchlorate, Nitrate & Thiocyanate	Thiocyanate	Total WBC	0.00 (0.00, 0.01)	0.002	0.005	0.02 (0.00, 0.04)	0.04	0.02 (0.00, 0.04)	0.04	0.14 (0.05, 0.45)	0.03
VOC Metabolites	2MHA	Total WBC	0.00 (0.00, 0.00)	0.004	0.008	0.02 (0.01, 0.04)	0.007	0.02 (0.01, 0.04)	0.007	0.10 (0.03, 0.26)	0.02
VOC Metabolites	3&4 MHA	Total WBC	0.00 (0.00, 0.00)	0.02	0.04	-0.01 (-0.02, 0.01)	0.30	-0.01 (-0.02, 0.01)	0.30	-0.13 (-1.23, 1.93)	0.41
VOC Metabolites	AAMA	Total WBC	-0.00 (-0.00, -0.00)	0.002	0.005	-0.00 (-0.01, 0.01)	0.81	-0.00 (-0.01, 0.01)	0.81	0.42 (-2.18, 3.21)	0.71
VOC Metabolites	AMCC	Total WBC	-0.00 (-0.00, 0.00)	0.18	0.23	-0.01 (-0.01, -0.00)	0.06	-0.01 (-0.01, -0.00)	0.06	0.04 (-0.03, 0.20)	0.30
VOC Metabolites	ATCA	Total WBC	0.00 (0.00, 0.01)	0.002	0.005	0.02 (0.01, 0.04)	0.007	0.02 (0.01, 0.04)	0.007	0.13 (0.04, 0.32)	0.02
VOC Metabolites	BMA	Total WBC	0.00 (0.00, 0.00)	0.004	0.008	0.01 (0.01, 0.02)	<0.001	0.01 (0.01, 0.02)	<0.001	0.14 (0.05, 0.31)	0.02
VOC Metabolites	BPMA	Total WBC	0.00 (0.00, 0.01)	0.002	0.005	0.02 (-0.00, 0.04)	0.14	0.02 (-0.00, 0.04)	0.14	0.10 (0.01, 0.57)	0.11
VOC Metabolites	CEMA	Total WBC	0.00 (0.00, 0.00)	<0.001	<0.001	0.01 (-0.00, 0.02)	0.24	0.01 (-0.00, 0.02)	0.24	0.18 (-0.58, 1.29)	0.16
VOC Metabolites	CYMA	Total WBC	0.00 (0.00, 0.00)	0.004	0.008	0.02 (0.01, 0.03)	<0.001	0.02 (0.01, 0.03)	<0.001	0.13 (0.04, 0.31)	0.02
VOC Metabolites	DHBMA	Total WBC	0.00 (0.00, 0.01)	0.002	0.005	0.02 (0.00, 0.04)	0.04	0.02 (0.00, 0.04)	0.04	0.12 (0.04, 0.42)	0.03
VOC Metabolites	2HPMA	Total WBC	0.00 (0.00, 0.01)	0.004	0.008	0.03 (0.01, 0.05)	0.007	0.03 (0.01, 0.05)	0.007	0.16 (0.05, 0.36)	0.02

© 2024 Guo J et al. *JAMA Network Open*.

VOC Metabolites	3HPMA	Total WBC	0.00 (0.00, 0.00)	<0.001	<0.001	0.01 (-0.01, 0.03)	0.32	0.01 (-0.01, 0.03)	0.32	0.16 (-1.25, 1.47)	0.23
VOC Metabolites	MA	Total WBC	0.00 (0.00, 0.00)	0.002	0.005	0.02 (0.01, 0.03)	0.007	0.02 (0.01, 0.03)	0.007	0.13 (0.05, 0.30)	0.02
VOC Metabolites	MHBMA3	Total WBC	0.00 (0.00, 0.00)	0.01	0.02	0.01 (0.00, 0.01)	<0.001	0.01 (0.00, 0.01)	<0.001	0.12 (0.03, 0.23)	0.04
VOC Metabolites	PGA	Total WBC	0.00 (0.00, 0.00)	0.01	0.02	0.01 (0.01, 0.01)	<0.001	0.01 (0.01, 0.01)	<0.001	0.09 (0.02, 0.17)	0.04
VOC Metabolites	HPMMA	Total WBC	0.00 (0.00, 0.00)	0.01	0.02	0.01 (0.01, 0.02)	<0.001	0.01 (0.01, 0.02)	<0.001	0.09 (0.02, 0.17)	0.04

Abbreviations: 2,2DCVMA, N-acetyl-S-(2,2-dichlorovinyl)-L-cysteine; AAMA, 2-carbamoylethylmercapturic acid; AMCC, methylcarbamoylmercapturate; ATCA, 2-aminothiazoline-4-carboxylic acid; BMA, benzylmercapturic acid; BPMA, N-acetyl-S-(n-propyl)-L-cysteine; CEMA, N-acetyl-S-(2-carboxyethyl)-L-cysteine; CYMA, N-acetyl-S-(2-cyanoethyl)-L-cysteine; DHBMA, dihydroxy-butyl-mercapturic acid; ExWAS, exposome-wide association study; GAMA, carbamoyl-2-hydroxyethylmercapturate; HEMA, 2-hydroxyethylmercapturic acid; HPMA, hydroxypropylmercapturic acid; HPMMA, 3-hydroxy-1-methyl-propylmercapturic acid; MA, mandelic acid; MHA, methylhippuric acid; MHBMA, N-acetyl-S-(2-hydroxy-3-butenyl)-L-cysteine; CI, confidence interval; PAH, polycyclic aromatic hydrocarbon; PGA, polyglutamic acid; PHEMA, poly(2-hydroxyethyl methacrylate); PMA, phenylmercapturic acid; TNE-2, total nicotine equivalent-2; VOC, volatile organic compound; WBC, white blood cell.

Mediation models were performed, adjusting for sex, age, race/Hispanic origin, education, the ratio of family income to poverty, BMI, sleep duration, alcohol consumption, estimated glomerular filtration rate, sample collection time, six-month survey period, and survey cycles. Estimates with 95% confidence intervals were derived by multiplying the initial values by a factor of 100.

* P values were adjusted to control the false discovery rate at 5%.

© 2024 Guo J et al. *JAMA Network Open*.

eTable 7. Mediation of the Inverse Association Between Environmental Toxicants and Depressive Symptoms by Inflammation Biomarkers

Exposure family	Exposure	Mediators	Indirect effect			Total effect		Direct effect		Proportion of mediation	
			Estimate (95% CI)	P	P*	Estimate (95% CI)	P*	Estimate (95% CI)	P*	Estimate (95% CI)	P*
Acrylamide & Glycidamide	Acrylamide	Total WBC	0.00 (0.00, 0.00)	0.008	0.01	0.01 (0.00, 0.01)	<0.001	0.01 (0.00, 0.01)	<0.001	0.11 (0.03, 0.28)	0.03
Acrylamide & Glycidamide	Glycidamide	Total WBC	0.00 (0.00, 0.00)	0.008	0.01	0.00 (0.00, 0.01)	0.09	0.00 (0.00, 0.01)	0.09	0.21 (0.05, 0.80)	0.05
Arsenics - Speciated	Dimethylarsinic Acid	Total WBC	0.00 (0.00, 0.00)	0.008	0.01	0.00 (-0.00, 0.00)	0.21	0.00 (-0.00, 0.00)	0.21	0.22 (-0.22, 1.52)	0.11
Ethylene Oxide	Ethylene Oxide	Total WBC	0.00 (-0.00, 0.00)	0.11	0.15	-0.00 (-0.00, 0.00)	0.79	-0.00 (-0.00, 0.00)	0.79	-0.48 (-1.82, 1.11)	0.91
Formaldehyde	Formaldehyde	Total WBC	-0.00 (-0.00, 0.00)	1.00	1.00	-0.00 (-0.00, 0.00)	0.43	-0.00 (-0.00, 0.00)	0.43	0.00 (-0.36, 0.45)	0.98
Iodine	Iodine	Total WBC	-0.00 (-0.00, 0.00)	0.35	0.41	0.00 (-0.00, 0.00)	0.21	0.00 (-0.00, 0.00)	0.21	-0.04 (-0.36, 0.24)	0.55
Metals	Copper	Total WBC	0.00 (0.00, 0.00)	0.008	0.01	0.02 (0.01, 0.03)	<0.001	0.02 (0.01, 0.03)	<0.001	0.10 (0.03, 0.20)	0.03
Metals	Selenium	Total WBC	-0.00 (-0.00, -0.00)	0.008	0.01	-0.01 (-0.01, 0.00)	0.11	-0.01 (-0.01, 0.00)	0.11	0.11 (0.00, 0.70)	0.10
Metals	Zinc	Total WBC	-0.00 (-0.00, 0.00)	0.06	0.09	-0.00 (-0.01, 0.00)	0.38	-0.00 (-0.01, 0.00)	0.38	0.10 (-0.65, 1.01)	0.43
Metals	Barium	Total WBC	0.00 (0.00, 0.00)	0.008	0.01	0.01 (-0.00, 0.02)	0.21	0.01 (-0.00, 0.02)	0.21	0.20 (-0.80, 1.35)	0.18
Metals	Cadmium	Total WBC	0.00 (0.00, 0.00)	0.008	0.01	0.03 (0.02, 0.04)	<0.001	0.03 (0.02, 0.04)	<0.001	0.07 (0.02, 0.15)	0.03
Metals	Cobalt	Total WBC	0.00 (0.00, 0.00)	0.008	0.01	0.01 (0.00, 0.01)	0.04	0.01 (0.00, 0.01)	0.04	0.16 (0.04, 0.46)	0.05
Metals	Cesium	Total WBC	0.00 (0.00, 0.00)	0.008	0.01	0.01 (0.00, 0.02)	0.04	0.01 (0.00, 0.02)	0.04	0.12 (0.03, 0.36)	0.05
Metals	Molybdenum	Total WBC	0.00 (0.00, 0.00)	0.008	0.01	0.01 (0.01, 0.02)	<0.001	0.01 (0.01, 0.02)	<0.001	0.11 (0.04, 0.26)	0.03
Metals	Lead	Total WBC	0.00 (0.00, 0.00)	0.008	0.01	0.00 (-0.00, 0.01)	0.21	0.00 (-0.00, 0.01)	0.21	0.18 (-0.52, 1.08)	0.18
Metals	Antimony	Total WBC	0.00 (0.00, 0.00)	0.008	0.01	0.00 (-0.00, 0.01)	0.24	0.00 (-0.00, 0.01)	0.24	0.16 (-1.52, 1.22)	0.23
Metals	Tin	Total WBC	0.00 (0.00, 0.00)	0.008	0.01	0.01 (-0.00, 0.01)	0.11	0.01 (-0.00, 0.01)	0.11	0.16 (0.02, 0.72)	0.09
Metals	Strontium	Total WBC	-0.00 (-0.00, 0.00)	0.07	0.10	-0.00 (-0.01, 0.00)	0.19	-0.00 (-0.01, 0.00)	0.19	0.06 (-0.29, 0.52)	0.24
Metals	Thallium	Total WBC	-0.00 (-0.00, 0.00)	0.97	0.99	-0.00 (-0.01, 0.00)	0.29	-0.00 (-0.01, 0.00)	0.29	0.00 (-0.35, 0.28)	0.98
Metals	Tungsten	Total WBC	0.00 (-0.00, 0.00)	0.22	0.28	-0.01 (-0.02, -0.00)	0.04	-0.01 (-0.02, -0.00)	0.04	-0.03 (-0.18, 0.02)	0.33
Metals	Uranium	Total WBC	0.00 (-0.00, 0.00)	0.72	0.75	0.00 (-0.00, 0.01)	0.21	0.00 (-0.00, 0.01)	0.21	0.01 (-0.29, 0.27)	0.84

© 2024 Guo J et al. *JAMA Network Open*.

Nicotine Metabolites	Total Cotinine	Total WBC	0.00 (-0.00, 0.00)	0.35	0.41	-0.00 (-0.01, 0.00)	0.42	-0.00 (-0.01, 0.00)	0.42	-0.06 (-0.63, 0.80)	0.70
Nicotine Metabolites	Total Hydroxycotinine	Total WBC	-0.00 (-0.00, 0.00)	0.09	0.12	-0.01 (-0.01, -0.00)	<0.001	-0.01 (-0.01, -0.00)	<0.001	0.03 (-0.00, 0.09)	0.16
Nicotine Metabolites	TNE-2	Total WBC	-0.00 (-0.00, 0.00)	0.42	0.46	-0.00 (-0.01, 0.00)	0.20	-0.00 (-0.01, 0.00)	0.20	0.03 (-0.18, 0.25)	0.57
PAH	1-napthol	Total WBC	-0.00 (-0.00, 0.00)	0.39	0.44	0.00 (-0.01, 0.01)	0.98	0.00 (-0.01, 0.01)	0.98	2.09 (-0.71, 1.10)	0.97
PAH	2-napthol	Total WBC	0.00 (-0.00, 0.00)	0.44	0.46	0.00 (-0.00, 0.01)	0.21	0.00 (-0.00, 0.01)	0.21	0.02 (-0.17, 0.33)	0.58
PAH	3-hydroxyfluorene	Total WBC	0.00 (-0.00, 0.00)	0.37	0.42	0.02 (0.01, 0.02)	<0.001	0.02 (0.01, 0.02)	<0.001	0.01 (-0.01, 0.05)	0.47
PAH	2-hydroxyfluorene	Total WBC	0.00 (-0.00, 0.00)	0.13	0.17	-0.00 (-0.01, 0.00)	0.40	-0.00 (-0.01, 0.00)	0.40	-0.10 (-1.02, 1.02)	0.55
PAH	1-hydroxyphenanthrene	Total WBC	-0.00 (-0.00, -0.00)	0.04	0.06	-0.01 (-0.01, -0.01)	<0.001	-0.01 (-0.01, -0.01)	<0.001	0.03 (0.00, 0.07)	0.08
PAH	1-hydroxypyrene	Total WBC	0.00 (-0.00, 0.00)	0.23	0.28	0.00 (-0.00, 0.01)	0.21	0.00 (-0.00, 0.01)	0.21	0.04 (-0.18, 0.44)	0.43
PAH	2&3-Hydroxyphenanthrene	Total WBC	0.00 (0.00, 0.00)	0.03	0.05	0.01 (0.00, 0.01)	0.08	0.01 (0.00, 0.01)	0.08	0.06 (-0.01, 0.38)	0.13
Perchlorate, Nitrate & Thiocyanate	Perchlorate	Total WBC	0.00 (0.00, 0.00)	0.008	0.01	0.01 (-0.00, 0.02)	0.10	0.01 (-0.00, 0.02)	0.10	0.13 (0.03, 0.72)	0.08
Perchlorate, Nitrate & Thiocyanate	Nitrate	Total WBC	0.00 (0.00, 0.00)	0.008	0.01	0.01 (0.01, 0.02)	0.007	0.01 (0.01, 0.02)	0.007	0.08 (0.02, 0.23)	0.03
Perchlorate, Nitrate & Thiocyanate	Thiocyanate	Total WBC	0.00 (0.00, 0.00)	0.008	0.01	0.01 (0.00, 0.01)	0.04	0.01 (0.00, 0.01)	0.04	0.12 (0.03, 0.45)	0.03
VOC Metabolites	2MHA	Total WBC	0.00 (0.00, 0.00)	0.008	0.01	0.01 (0.00, 0.01)	0.04	0.01 (0.00, 0.01)	0.04	0.12 (0.03, 0.42)	0.06
VOC Metabolites	3&4 MHA	Total WBC	0.00 (0.00, 0.00)	0.02	0.04	-0.01 (-0.01, 0.00)	0.11	-0.01 (-0.01, 0.00)	0.11	-0.06 (-0.48, 0.18)	0.17
VOC Metabolites	AAMA	Total WBC	-0.00 (-0.00, -0.00)	0.01	0.02	-0.00 (-0.01, 0.00)	0.50	-0.00 (-0.01, 0.00)	0.50	0.19 (-1.36, 2.05)	0.47
VOC Metabolites	AMCC	Total WBC	-0.00 (-0.00, 0.00)	0.25	0.30	-0.01 (-0.02, -0.00)	0.01	-0.01 (-0.02, -0.00)	0.01	0.02 (-0.02, 0.09)	0.35
VOC Metabolites	ATCA	Total WBC	0.00 (0.00, 0.00)	0.008	0.01	0.01 (0.01, 0.02)	<0.001	0.01 (0.01, 0.02)	<0.001	0.07 (0.02, 0.16)	0.03
VOC Metabolites	BMA	Total WBC	0.00 (0.00, 0.01)	0.008	0.01	0.04 (0.02, 0.05)	<0.001	0.04 (0.02, 0.05)	<0.001	0.09 (0.03, 0.18)	0.03
VOC Metabolites	BPMA	Total WBC	0.00 (0.00, 0.00)	0.01	0.02	0.00 (0.00, 0.01)	0.06	0.00 (0.00, 0.01)	0.06	0.07 (0.01, 0.34)	0.08
VOC Metabolites	CEMA	Total WBC	0.00 (0.00, 0.00)	0.008	0.01	0.01 (0.00, 0.02)	0.03	0.01 (0.00, 0.02)	0.03	0.08 (0.02, 0.27)	0.04
VOC Metabolites	CYMA	Total WBC	0.00 (0.00, 0.00)	0.008	0.01	0.01 (0.00, 0.02)	0.01	0.01 (0.00, 0.02)	0.01	0.10 (0.03, 0.25)	0.03
VOC Metabolites	DHBMA	Total WBC	0.00 (0.00, 0.00)	0.008	0.01	0.01 (0.00, 0.01)	0.01	0.01 (0.00, 0.01)	0.01	0.08 (0.02, 0.25)	0.03
VOC Metabolites	2HPMA	Total WBC	0.00 (0.00, 0.00)	0.008	0.01	0.02 (0.01, 0.02)	<0.001	0.02 (0.01, 0.02)	<0.001	0.11 (0.04, 0.24)	0.03

© 2024 Guo J et al. *JAMA Network Open*.

VOC Metabolites	3HPMA	Total WBC	0.00 (0.00, 0.00)	0.008	0.01	0.00 (-0.00, 0.01)	0.21	0.00 (-0.00, 0.01)	0.21	0.11 (-0.68, 0.93)	0.18
VOC Metabolites	MA	Total WBC	0.00 (0.00, 0.00)	0.008	0.01	0.01 (0.00, 0.02)	<0.001	0.01 (0.00, 0.02)	<0.001	0.10 (0.03, 0.25)	0.03
VOC Metabolites	MHBMA3	Total WBC	0.01 (0.00, 0.01)	0.008	0.01	0.08 (0.05, 0.11)	<0.001	0.08 (0.05, 0.11)	<0.001	0.09 (0.03, 0.17)	0.03
VOC Metabolites	PGA	Total WBC	0.01 (0.00, 0.01)	0.008	0.01	0.09 (0.06, 0.12)	<0.001	0.09 (0.06, 0.12)	<0.001	0.07 (0.02, 0.14)	0.03
VOC Metabolites	HPMMA	Total WBC	0.01 (0.00, 0.01)	0.008	0.01	0.09 (0.06, 0.12)	<0.001	0.09 (0.06, 0.12)	<0.001	0.07 (0.02, 0.14)	0.03

Abbreviations: 2,2DCVMA, N-acetyl-S-(2,2-dichlorovinyl)-L-cysteine; AAMA, 2-carbamoylethylmercapturic acid; AMCC, methylcarbamoylmercapturate; ATCA, 2-aminothiazoline-4-carboxylic acid; BMA, benzylmercapturic acid; BPMA, N-acetyl-S-(n-propyl)-L-cysteine; CEMA, N-acetyl-S-(2-carboxyethyl)-L-cysteine; CYMA, N-acetyl-S-(2-cyanoethyl)-L-cysteine; DHBMA, dihydroxy-butyl-mercapturic acid; ExWAS, exposome-wide association study; GAMA, carbamoyl-2-hydroxyethylmercapturate; HEMA, 2-hydroxyethylmercapturic acid; HPMA, hydroxypropylmercapturic acid; HPMMA, 3-hydroxy-1-methyl-propylmercapturic acid; MA, mandelic acid; MHA, methylhippuric acid; MHBMA, N-acetyl-S-(2-hydroxy-3-butenyl)-L-cysteine; CI, confidence interval; PAH, polycyclic aromatic hydrocarbon; PGA, polyglutamic acid; PHEMA, poly(2-hydroxyethyl methacrylate); PMA, phenylmercapturic acid; TNE-2, total nicotine equivalent-2; VOC, volatile organic compound; WBC, white blood cell.

Mediation models were performed, adjusting for sex, age, race/Hispanic origin, education, the ratio of family income to poverty, BMI, sleep duration, alcohol consumption, estimated glomerular filtration rate, sample collection time, six-month survey period, and survey cycles. Estimates with 95% confidence intervals were derived by multiplying the initial values by a factor of 100.

* P values were adjusted to control the false discovery rate at 5%.

© 2024 Guo J et al. *JAMA Network Open*.

eTable 8. Mediation of the Association Between Exposure-Class Risk Score (ERS) and Depressive Symptoms by Inflammation Biomarkers

Exposure family	Mediators	Indirect effect			Total effect		Direct effect		Proportion of mediation	
		**Estimate (95% CI)**	**P**	**P***	**Estimate (95% CI)**	**P***	**Estimate (95% CI)**	**P***	**Estimate (95% CI)**	**P***
Metals	Total WBC	0.00 (0.00, 0.01)	<0.001	<0.001	1.00 (0.96, 1.00)	<0.001	1.00 (0.96, 1.00)	<0.001	0.00 (0.00, 0.01)	<0.001
Perchlorate, Nitrate & Thiocyanate	Total WBC	-0.03 (-0.11, -0.01)	<0.001	<0.001	-0.05 (-0.06, 0.06)	0.11	-0.05 (-0.06, 0.06)	0.11	0.37 (0.16, 1.34)	0.04
PAH	Total WBC	0.03 (0.01, 0.07)	0.008	0.008	0.90 (0.82, 0.93)	<0.001	0.90 (0.82, 0.93)	<0.001	0.03 (0.01, 0.07)	0.01
VOC Metabolites	Total WBC	0.04 (0.01, 0.11)	0.006	0.008	0.86 (0.71, 0.91)	<0.001	0.86 (0.71, 0.91)	<0.001	0.04 (0.01, 0.13)	0.01
Nicotine Metabolites	Total WBC	0.06 (0.02, 0.13)	0.008	0.008	0.81 (0.72, 0.85)	<0.001	0.81 (0.72, 0.85)	<0.001	0.07 (0.02, 0.15)	0.01

Abbreviations: CI, confidence interval; WBC, white blood cell; PAH, polycyclic aromatic hydrocarbon; VOC, volatile organic compound.
Mediation models were performed, adjusting for sex, age, race/Hispanic origin, education, the ratio of family income to poverty, BMI, sleep duration, alcohol consumption, estimated glomerular filtration rate, sample collection time, six-month survey period, and survey cycles. Estimates with 95% confidence intervals were derived by multiplying the initial values by a factor of 100.
* P values were adjusted to control the false discovery rate at 5%.

© 2024 Guo J et al. *JAMA Network Open*.

eFigure 1. Flowchart of Sample Selection

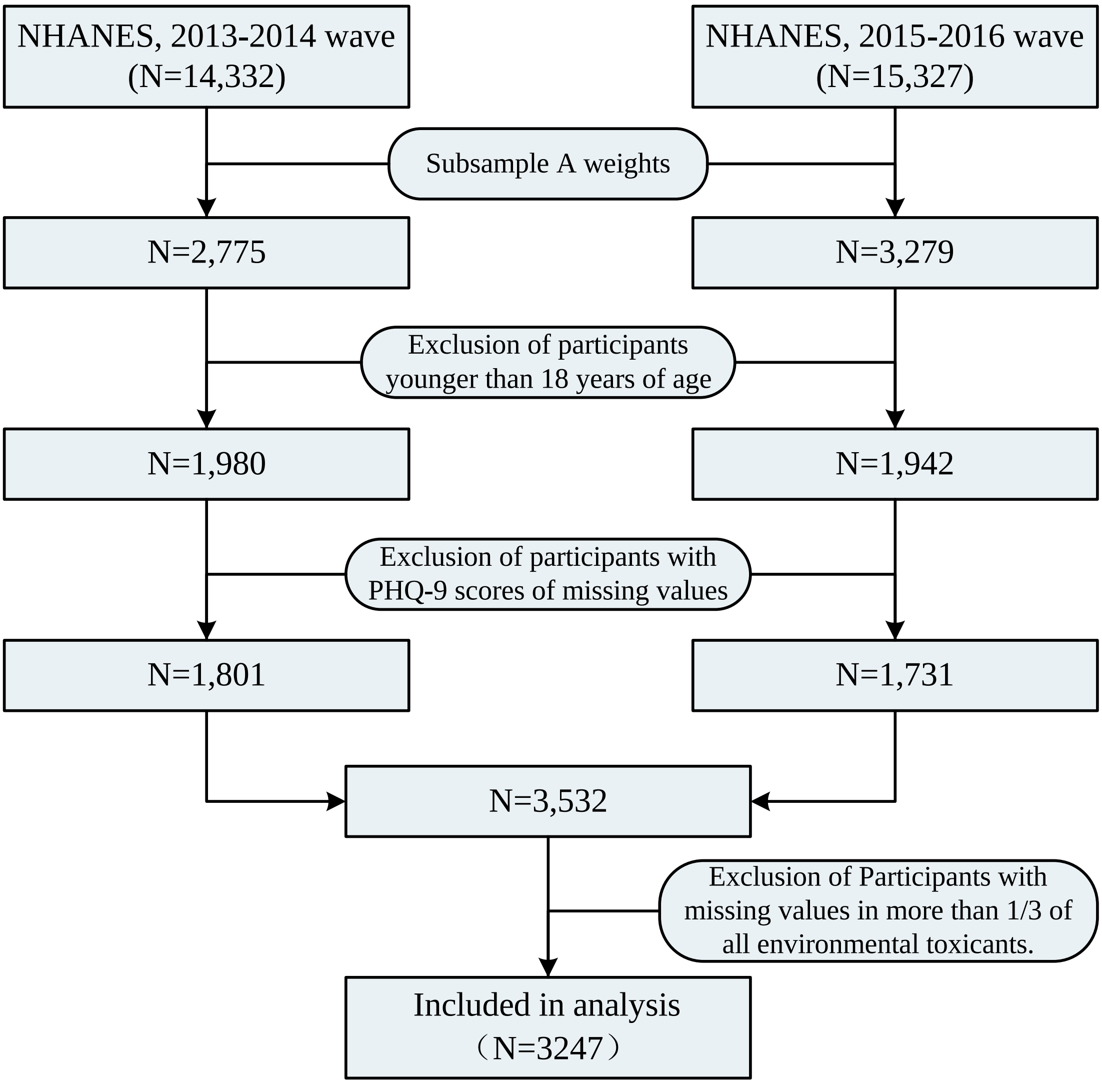

© 2024 Guo J et al. *JAMA Network Open*.

eFigure 2. Correlation of the Environmental Toxicants (62 Exposures)

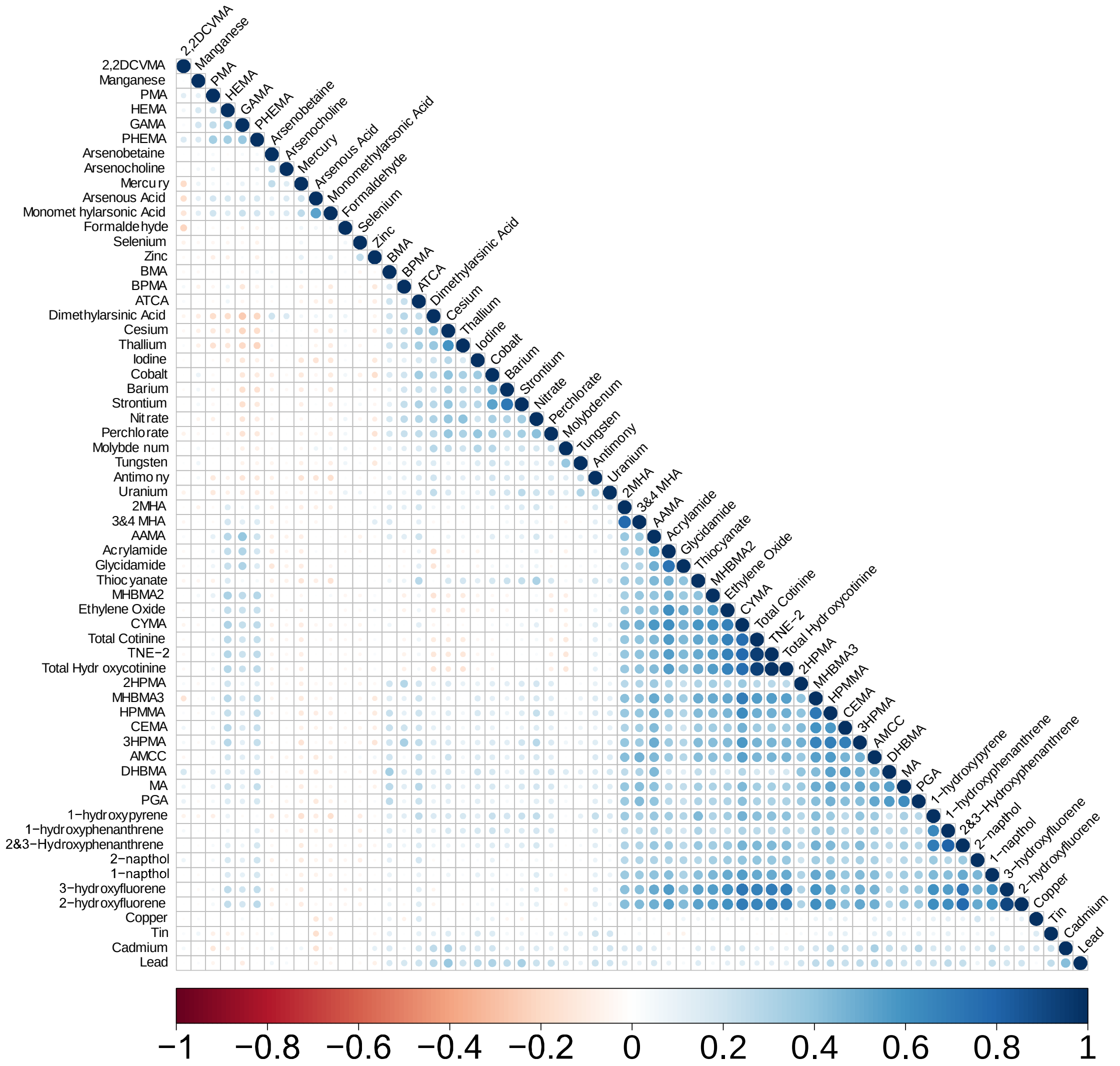

Abbreviations: 2,2DCVMA, N-acetyl-S-(2,2-dichlorovinyl)-L-cysteine; AAMA, 2-carbamoylethylmercapturic acid; AMCC, methylcarbamoylmercapturate; ATCA, 2-aminothiazoline-4-carboxylic acid; BMA, benzylmercapturic acid; BPMA, N-acetyl-S-(n-propyl)-L-cysteine; CEMA, N-acetyl-S-(2-carboxyethyl)-L-cysteine; CYMA, N-acetyl-S-(2-cyanoethyl)-L-cysteine; DHBMA, dihydroxy-butyl-mercapturic acid; ExWAS, exposome-wide association study; GAMA, carbamoyl-2-hydroxyethylmercapturate; HEMA, 2-hydroxyethylmercapturic acid; HPMA, hydroxypropylmercapturic acid; HPMMA, 3-hydroxy-1-methyl-propylmercapturic acid; MA, mandelic acid; MHA, methylhippuric acid; MHBMA, N-acetyl-S-(2-hydroxy-3-butenyl)-L-cysteine; PGA, polyglutamic acid; PHEMA, poly(2-hydroxyethyl methacrylate); PMA, phenylmercapturic acid; TNE-2, total nicotine equivalent-2.

© 2024 Guo J et al. *JAMA Network Open.*

eFigure 3. Adjusted Associations Between the Environmental Exposome (62 Exposures) and Depressive Symptoms in 3427 Adults (ExWAS Analysis), Compared With Threshold-Based Results

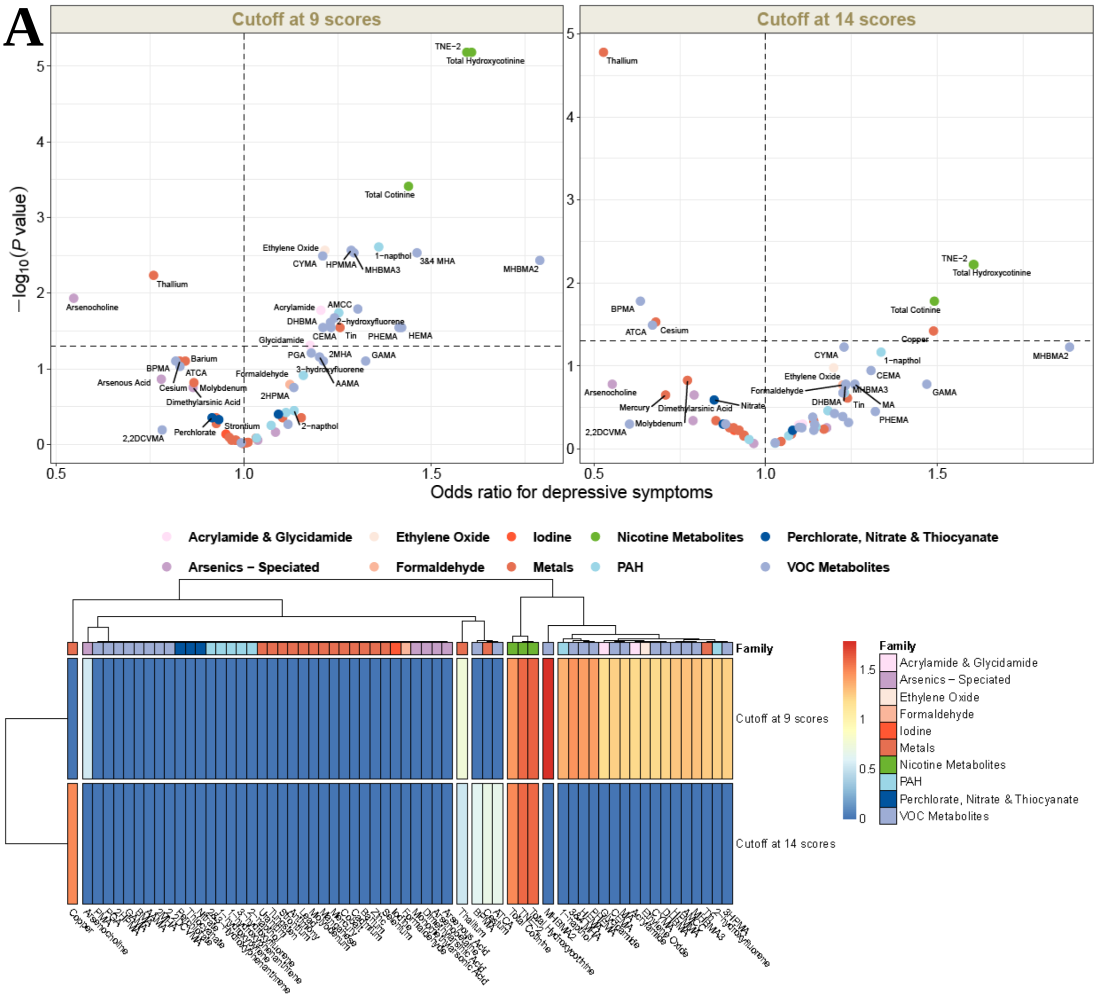

Abbreviations: 2,2DCVMA, N-acetyl-S-(2,2-dichlorovinyl)-L-cysteine; AAMA, 2-carbamoylethylmercapturic acid; AMCC, methylcarbamoylmercapturate; ATCA, 2-aminothiazoline-4-carboxylic acid; BMA, benzylmercapturic acid; BPMA, N-acetyl-S-(n-propyl)-L-cysteine; CEMA, N-acetyl-S-(2-carboxyethyl)-L-cysteine; CYMA, N-acetyl-S-(2-cyanoethyl)-L-cysteine; DHBMA, dihydroxy-butyl-mercapturic acid; ExWAS, exposome-wide association study; GAMA, carbamoyl-2-hydroxyethylmercapturate; HEMA, 2-hydroxyethylmercapturic acid; HPMA, hydroxypropylmercapturic acid; HPMMA, 3-hydroxy-1-methyl-propylmercapturic acid; MA, mandelic acid; MHA, methylhippuric acid; MHBMA, N-acetyl-S-(2-hydroxy-3-butenyl)-L-cysteine; PAH, polycyclic aromatic hydrocarbon; PGA, polyglutamic acid; PHEMA, poly(2-hydroxyethyl methacrylate); PMA, phenylmercapturic acid; TNE-2, total nicotine equivalent-2; VOC, volatile organic compound.

Panel A presents the stratified results of the ExWAS analyses by year of survey. Panel

© 2024 Guo J et al. *JAMA Network Open*.

B presents a heatmap and clustered relationships indicating the ORs of environmental toxicants among various subgroups, and if the P value was >0.05 for the OR, the OR was replaced by 0.
ExWAS analyses were performed, adjusting for sex, age, race/Hispanic origin, education, the ratio of family income to poverty, BMI, sleep duration, alcohol consumption, estimated glomerular filtration rate, sample collection time, six-month survey period, and survey cycles.
The p value was adjusted to control the false discovery rate at 5%.

© 2024 Guo J et al. *JAMA Network Open*.

eFigure 4. Sensitivity Analyses of Adjusted Associations Between the Environmental Exposome (62 Exposures) and Depressive Symptoms (ExWAS Analysis), Compared With Threshold-Based Results

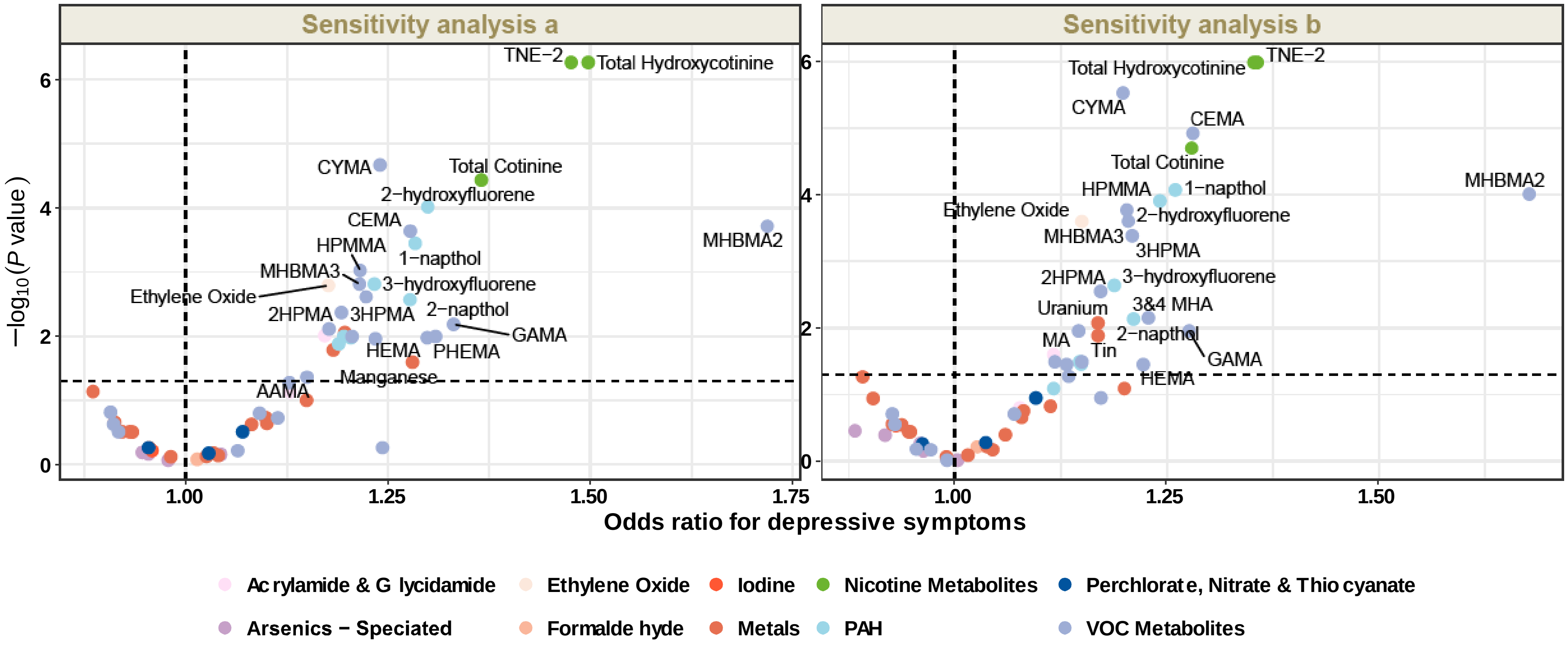

Abbreviations: 2,2DCVMA, N-acetyl-S-(2,2-dichlorovinyl)-L-cysteine; AAMA, 2-carbamoylethylmercapturic acid; AMCC, methylcarbamoylmercapturate; ATCA, 2-aminothiazoline-4-carboxylic acid; BMA, benzylmercapturic acid; BPMA, N-acetyl-S-(n-propyl)-L-cysteine; CEMA, N-acetyl-S-(2-carboxyethyl)-L-cysteine; CYMA, N-acetyl-S-(2-cyanoethyl)-L-cysteine; DHBMA, dihydroxy-butyl-mercapturic acid; ExWAS, exposome-wide association study; GAMA, carbamoyl-2-hydroxyethylmercapturate; HEMA, 2-

© 2024 Guo J et al. *JAMA Network Open*.

hydroxyethylmercapturic acid; HPMA, hydroxypropylmercapturic acid; HPMMA, 3-hydroxy-1-methyl-propylmercapturic acid; MA, mandelic acid; MHA, methylhippuric acid; MHBMA, N-acetyl-S-(2-hydroxy-3-butenyl)-L-cysteine; PAH, polycyclic aromatic hydrocarbon; PGA, polyglutamic acid; PHEMA, poly(2-hydroxyethyl methacrylate); PMA, phenylmercapturic acid; TNE-2, total nicotine equivalent-2; VOC, volatile organic compound.

ExWAS analyses were performed, adjusting for sex, age, race/Hispanic origin, education, the ratio of family income to poverty, BMI, sleep duration, alcohol consumption, estimated glomerular filtration rate, sample collection time, six-month survey period, and survey cycles.

Sensitivity analysis a was conducted based on data that did not impute missing values.

Sensitivity analysis b was conducted based on data that did not exclude participants with missing values for more than 1/3 of all environmental toxicants.

The p value was adjusted to control the false discovery rate at 5%.

© 2024 Guo J et al. *JAMA Network Open*.

eFigure 5. Mediation of the Inverse Association Between Environmental Toxicants and Depressive Symptoms by Total WBC by Pairwise Mediation Analysis

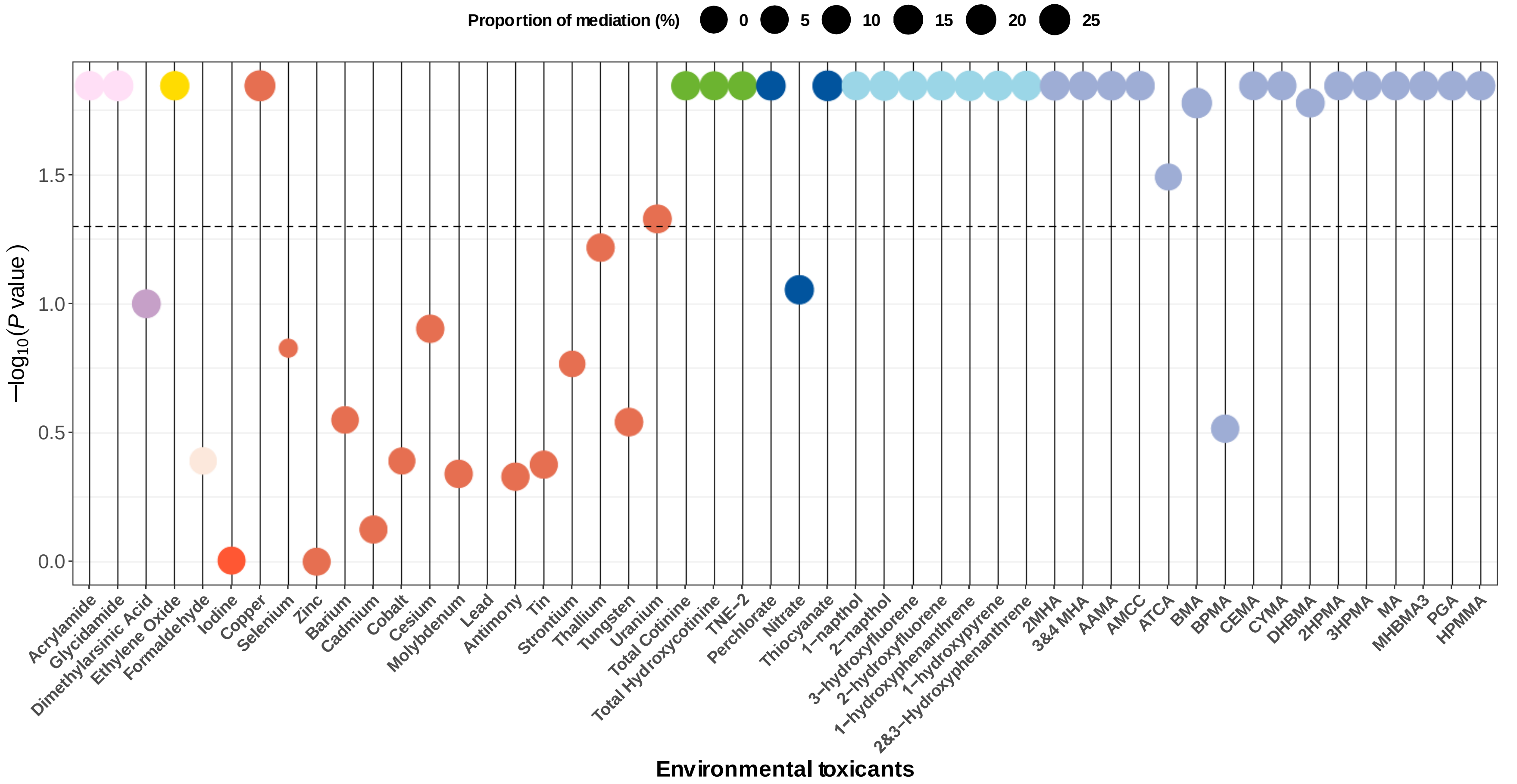

© 2024 Guo J et al. *JAMA Network Open*.

Abbreviations: 2,2DCVMA, N-acetyl-S-(2,2-dichlorovinyl)-L-cysteine; AAMA, 2-carbamoylethylmercapturic acid; AMCC, methylcarbamoylmercapturate; ATCA, 2-aminothiazoline-4-carboxylic acid; BMA, benzylmercapturic acid; BPMA, N-acetyl-S-(n-propyl)-L-cysteine; CEMA, N-acetyl-S-(2-carboxyethyl)-L-cysteine; CYMA, N-acetyl-S-(2-cyanoethyl)-L-cysteine; DHBMA, dihydroxy-butyl-mercapturic acid; ExWAS, exposome-wide association study; GAMA, carbamoyl-2-hydroxyethylmercapturate; HEMA, 2-hydroxyethylmercapturic acid; HPMA, hydroxypropylmercapturic acid; HPMMA, 3-hydroxy-1-methyl-propylmercapturic acid; MA, mandelic acid; MHA, methylhippuric acid; MHBMA, N-acetyl-S-(2-hydroxy-3-butenyl)-L-cysteine; PHEMA, poly(2-hydroxyethyl methacrylate); PMA, phenylmercapturic acid; TNE-2, total nicotine equivalent-2; WBC, white blood cell.

The red line serves as a reference line, indicating $y = -\log_{10}(0.05)$.

Mediation models were performed, adjusting sex, age, race/Hispanic origin, education, ratio of family income to poverty, BMI, sleep duration, alcohol consumption, estimate glomerular filtration rate, sample collection time, six-month survey period, and survey cycles.

P-value have been adjusted to control the false discovery rate at 5%.

© 2024 Guo J et al. *JAMA Network Open*.

eReferences

1. Centers for Disease Control and Prevention (CDC). Laboratory Data Overview of NHANES 2013-2014. https://wwwn.cdc.gov/nchs/nhanes/continuousnhanes/overviewlab.aspx?BeginYear=2013. Accessed December 28, 2023.
2. Centers for Disease Control and Prevention (CDC). Laboratory Data Overview of NHANES 2015-2016. https://wwwn.cdc.gov/nchs/nhanes/continuousnhanes/overviewlab.aspx?BeginYear=2015. Accessed December 28, 2023.
3. Centers for Disease Control and Prevention (CDC). Acrylamide, Glycidamide and Ethylene Oxide Lab Procedure Manual. https://wwwn.cdc.gov/nchs/data/nhanes/2013-2014/labmethods/AMDGYD_ETHOX_H_MET.pdf. Accessed December 28, 2023.
4. Pala M, Ugolini D, Ceppi M, Rizzo F, Maiorana L, Bolognesi C, Schilirò T, Gilli G, Bigatti P, Bono R, Vecchio D. Occupational exposure to formaldehyde and biological monitoring of Research Institute workers. *Cancer Detect Prev*. 2008;32(2):121-6.
5. Metz B, Kersten GF, Baart GJ, de Jong A, Meiring H, ten Hove J, van Steenbergen MJ, Hennink WE, Crommelin DJ, Jiskoot W. Identification of formaldehyde-induced modifications in proteins: reactions with insulin. *Bioconjug Chem*. May-Jun 2006;17(3):815-22.
6. Centers for Disease Control and Prevention (CDC). Formaldehyde Lab Procedure Manual. https://wwwn.cdc.gov/nchs/data/nhanes/2013-2014/labmethods/FORMAL-FORMAS-H-MET-508.pdf. Accessed December 28, 2023.
7. Centers for Disease Control and Prevention (CDC). Aromatic Amines Laboratory Procedure Manual. https://wwwn.cdc.gov/nchs/data/nhanes/2013-2014/labmethods/AA-AAS-H-MET-508.pdf. Accessed December 28, 2023.
8. Centers for Disease Control and Prevention (CDC). Arsenobetaine, Arsenocholine,

© 2024 Guo J et al. *JAMA Network Open*.

Trimethylarsine Oxide, Monomethylarsonic Acid, Dimethylarsinic Acid, Arsenous (III) Acid, Arsenic (V) Acid Laboratory Procedure Manual. https://wwwn.cdc.gov/nchs/data/nhanes/2013-2014/labmethods/UAS_UASS_H_MET.pdf. Accessed December 28, 2023.

9. Centers for Disease Control and Prevention (CDC). URINARY METALS AND TOTAL ARSENIC Laboratory Procedure Manual. https://wwwn.cdc.gov/nchs/data/nhanes/2013-2014/labmethods/UM_UMS_UTAS_UTASS_H_MET.pdf. Accessed December 28, 2023.

10. Centers for Disease Control and Prevention (CDC). Urinary Cotinine and Hydroxycotinine Laboratory Procedure Manual. https://wwwn.cdc.gov/nchs/data/nhanes/2013-2014/labmethods/UCOT_H_UCOTS_H_Urinary_Cotinine_and_Hydroxycotinine_MET.pdf. Accessed December 28, 2023.

11. Centers for Disease Control and Prevention (CDC). Urinary Nicotine Metabolites and Analogs Laboratory Procedure Manual. https://wwwn.cdc.gov/nchs/data/nhanes/2013-2014/labmethods/UCOT_H_UCOTS_H_Urinary_Nicotine_Metabolites_and_Analogs.pdf. Accessed December 28, 2023.

12. Centers for Disease Control and Prevention (CDC). Urinary Perchlorate, Nitrate, and Thiocyanate Laboratory Procedure Manual. https://wwwn.cdc.gov/nchs/data/nhanes/2013-2014/labmethods/PERNT-H-PERNTS-H-U1PN-H-R-U2PN-H-R-MET-508.pdf. Accessed December 28, 2023.

13. Wang Y, Meng L, Pittman EN, Etheredge A, Hubbard K, Trinidad DA, Kato K, Ye X, Calafat AM. Quantification of urinary mono-hydroxylated metabolites of polycyclic aromatic hydrocarbons by on-line solid phase extraction-high performance liquid chromatography-tandem mass spectrometry. *Anal Bioanal Chem.* Feb 2017;409(4):931-937.

© 2024 Guo J et al. *JAMA Network Open*.

14. Centers for Disease Control and Prevention (CDC). Volatile N-Nitrosamine Compounds Laboratory Procedure Manual. https://wwwn.cdc.gov/nchs/data/nhanes/2013-2014/labmethods/VNA_VNAS_H_MET.pdf. Accessed December 28, 2023.
15. Alwis KU, Blount BC, Britt AS, Patel D, Ashley DL. Simultaneous analysis of 28 urinary VOC metabolites using ultra high performance liquid chromatography coupled with electrospray ionization tandem mass spectrometry (UPLC-ESI/MSMS). *Anal Chim Acta*. Oct 31 2012;750:152-60.
16. Boss J, Mukherjee B, Ferguson KK, Aker A, Alshawabkeh AN, Cordero JF, Meeker JD, Kim S. Estimating Outcome-Exposure Associations when Exposure Biomarker Detection Limits vary Across Batches. *Epidemiology*. Sep 2019;30(5):746-755.
17. Patel CJ, Ioannidis JP. Placing epidemiological results in the context of multiplicity and typical correlations of exposures. *J Epidemiol Community Health*. Nov 2014;68(11):1096-100.
18. Vrijheid M, Fossati S, Maitre L, Márquez S, Roumeliotaki T, Agier L, Andrusaityte S, Cadiou S, Casas M, de Castro M, Dedele A, Donaire-Gonzalez D, Grazuleviciene R, Haug LS, McEachan R, Meltzer HM, Papadopouplou E, Robinson O, Sakhi AK, Siroux V, Sunyer J, Schwarze PE, Tamayo-Uria I, Urquiza J, Vafeiadi M, Valentin A, Warembourg C, Wright J, Nieuwenhuijsen MJ, Thomsen C, Basagaña X, Slama R, Chatzi L. Early-Life Environmental Exposures and Childhood Obesity: An Exposome-Wide Approach. *Environ Health Perspect*. Jun 2020;128(6):67009.
19. Agier L, Basagaña X, Maitre L, Granum B, Bird PK, Casas M, Oftedal B, Wright J, Andrusaityte S, de Castro M, Cequier E, Chatzi L, Donaire-Gonzalez D, Grazuleviciene R, Haug LS, Sakhi AK, Leventakou V, McEachan R, Nieuwenhuijsen M, Petraviciene I, Robinson O, Roumeliotaki T, Sunyer J, Tamayo-Uria I, Thomsen C, Urquiza J, Valentin A, Slama R, Vrijheid M, Siroux V. Early-life exposome and lung function in children in Europe: an analysis of data from the longitudinal, population-

© 2024 Guo J et al. *JAMA Network Open*.

based HELIX cohort. *Lancet Planet Health*. Feb 2019;3(2):e81-e92.

20. Moccia C, Pizzi C, Moirano G, Popovic M, Zugna D, d'Errico A, Isaevska E, Fossati S, Nieuwenhuijsen MJ, Fariselli P, Sanavia T, Richiardi L, Maule M. Modelling socioeconomic position as a driver of the exposome in the first 18 months of life of the NINFEA birth cohort children. *Environ Int*. Mar 2023;173:107864.

21. Benjamini Y, Hochberg Y. Controlling the false discovery rate: a practical and powerful approach to multiple testing. *Journal of the Royal statistical society: series B (Methodological)*. 1995;57(1):289-300.

22. Tzoulaki I, Patel CJ, Okamura T, Chan Q, Brown IJ, Miura K, Ueshima H, Zhao L, Van Horn L, Daviglus ML, Stamler J, Butte AJ, Ioannidis JP, Elliott P. A nutrient-wide association study on blood pressure. *Circulation*. Nov 20 2012;126(21):2456-64.

23. Gelman A. Struggles with survey weighting and regression modeling. 2007;

24. Korn EL, Graubard BI. Epidemiologic studies utilizing surveys: accounting for the sampling design. *American journal of public health*. 1991;81(9):1166-1173.

25. James-Todd T, Stahlhut R, Meeker JD, Powell S-G, Hauser R, Huang T, Rich-Edwards J. Urinary phthalate metabolite concentrations and diabetes among women in the National Health and Nutrition Examination Survey (NHANES) 2001–2008. *Environmental health perspectives*. 2012;120(9):1307-1313.

26. Lewis RC, Johns LE, Meeker JD. Serum biomarkers of exposure to perfluoroalkyl substances in relation to serum testosterone and measures of thyroid function among adults and adolescents from NHANES 2011–2012. *International journal of environmental research and public health*. 2015;12(6):6098-6114.

27. Lewis RC, Meeker JD. Biomarkers of exposure to molybdenum and other metals in relation to testosterone among men from the United States National Health and Nutrition Examination Survey 2011–2012. *Fertility and sterility*. 2015;103(1):172-178.

28. Stahlhut RW, Van Wijngaarden E, Dye TD, Cook S, Swan SH. Concentrations of urinary phthalate metabolites are associated with increased waist circumference and insulin resistance in adult US males. *Environmental health perspectives*.

© 2024 Guo J et al. *JAMA Network Open*.

2007;115(6):876-882.

29. Sinisi SE, van der Laan MJ. Deletion/substitution/addition algorithm in learning with applications in genomics. *Stat Appl Genet Mol Biol*. 2004;3:Article18.

30. Nieuwenhuijsen MJ, Agier L, Basagaña X, Urquiza J, Tamayo-Uria I, Giorgis-Allemand L, Robinson O, Siroux V, Maitre L, de Castro M, Valentin A, Donaire D, Dadvand P, Aasvang GM, Krog NH, Schwarze PE, Chatzi L, Grazuleviciene R, Andrusaityte S, Dedele A, McEachan R, Wright J, West J, Ibarluzea J, Ballester F, Vrijheid M, Slama R. Influence of the Urban Exposome on Birth Weight. *Environ Health Perspect*. Apr 2019;127(4):47007.

31. Hayes AF, Preacher KJ. Statistical mediation analysis with a multicategorical independent variable. *Br J Math Stat Psychol*. Nov 2014;67(3):451-70.

32. Zou H, Zhang HH. ON THE ADAPTIVE ELASTIC-NET WITH A DIVERGING NUMBER OF PARAMETERS. *Ann Stat*. 2009;37(4):1733-1751.

33. Vieira CLZ, Garshick E, Alvares D, Schwartz J, Huang S, Vokonas P, Gold DR, Koutrakis P. Association between ambient beta particle radioactivity and lower hemoglobin concentrations in a cohort of elderly men. *Environ Int*. Jun 2020;139:105735.

34. van den Brand JA, van Boekel GA, Willems HL, Kiemeney LA, den Heijer M, Wetzels JF. Introduction of the CKD-EPI equation to estimate glomerular filtration rate in a Caucasian population. *Nephrol Dial Transplant*. Oct 2011;26(10):3176-81.

© 2024 Guo J et al. *JAMA Network Open*.
