# Supplementary material for: Environmental Toxicant Exposure and Depressive Symptoms
Source: JAMA Netw Open. 2024 Jul 3;7(7):e2420259. doi: 10.1001/jamanetworkopen.2024.20259 (PMC11222999; doi:10.1001/jamanetworkopen.2024.20259)
Supplement: Supplement 2. — Data Sharing Statement [file jamanetwopen-e2420259-s002.pdf]

Data Sharing Statement

Guo. Environmental Toxicant Exposure and Depressive Symptoms. *JAMA Netw Open*.
Published July 03, 2024. doi:10.1001/jamanetworkopen.2024.20259

Data
Data available: No
